# Local treatment of HVJ-E with T cell costimulatory molecule stimulation elicits systemic anti-tumor effects

**DOI:** 10.1016/j.omton.2024.200893

**Published:** 2024-10-10

**Authors:** Airi Ishibashi, Yue Li, Yuuta Hisatomi, Noriko Ohta, Yuko Uegaki, Atsushi Tanemura, Riuko Ohashi, Koji Kitamura, Kotaro Saga, Yasuhide Yoshimura, Satoko Inubushi, Kyoso Ishida, Sadahiro Iwabuchi, Shinichi Hashimoto, Eiji Kiyohara, Hideo Yagita, Yasufumi Kaneda, Keisuke Nimura

**Affiliations:** 1 Division of Gene Therapy Science, Department of Genome Biology, Osaka University Graduate School of Medicine, Suita, Osaka 565-0871, Japan; 2 Division of Gene Therapy Science, Gunma University Initiative for Advanced Research, Gunma University, Maebashi, Gunma 371-8511, Japan; 3 Department of Dermatology, Osaka University Graduate School of Medicine, Suita, Osaka 565-0871, Japan; 4 Division of Molecular and Diagnostic Pathology, Niigata University Graduate School of Medical and Dental Sciences, Niigata 951-8510, Japan; 5 Histopathology Core Facility, Center for Research Promotion, Niigata University School of Medicine, Niigata 951-8510, Japan; 6 Department of Otorhinolaryngology-Head and Neck Surgery, Osaka University Graduate School of Medicine, Suita, Osaka 565-0871, Japan; 7 Department of Obstetrics and Gynecology, Osaka University Graduate School of Medicine, Suita, Osaka 565-0871, Japan; 8 Department of Molecular Pathophysiology, Institute of Advanced Medicine, Wakayama Medical University, Wakayama 641-8509, Japan; 9 Department of Immunology, Juntendo University School of Medicine, Bunkyo-Ku, Tokyo 113-8421, Japan

**Keywords:** Sendai virus, HVJ, HVJ-E, OX40, Nkg2d, Nkg2d ligand, abscopal effects, 4-1BB, tumor immunity, local treatment

## Abstract

The tumor-infiltrating lymphocyte (TIL) is a crucial factor in controlling tumor growth. A therapeutic method activating TIL is desired for treating patients with metastatic tumors. Here, we show that treating a local tumor with a combination therapy of UV-irradiated hemagglutinating virus of Japan envelope (HVJ-E) plus agonist antibodies, including OX40, against T cell costimulatory molecules induces systemic anti-tumor effects in a T cell-dependent manner in multiple cancer cell lines. Transcriptome and T cell receptor repertoire analyses revealed that HVJ-E + anti-OX40 antibody treatment activates CD4 and CD8 T cells and promotes T cell trafficking between tumors. These systemic anti-tumor effects required an association between Nkg2d and Nkg2d ligands. Our findings provide insights into how systemic anti-tumor effects are induced and may help the development of therapeutic strategies for eliciting such effects.

## Graphical abstract

**Figure:**
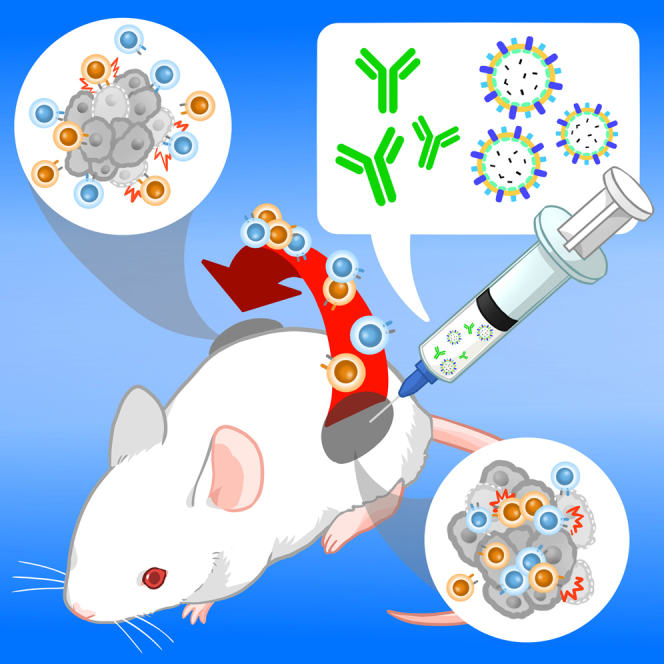

## Teaser

Nimura and colleagues found that local treatment with combination therapy of UV-irradiated hemagglutinating virus of Japan plus agonist antibodies against T cell costimulatory molecules induces systemic anti-tumor effects in a T cell-dependent manner. This therapy promotes T cell trafficking between tumors and relies on the interaction between Nkg2d and Nkg2d ligands.

## Introduction

Cancer immunotherapy aims to augment the quantity and activity of T cells capable of recognizing cancer cells in patients with tumors. Expanding T cells that identify cancer cells *in situ* is advantageous for avoiding cancer cell escape from T cell recognition and overcoming individual differences and cancer cell heterogeneity in each patient. Systemic administration of immune checkpoint inhibitors targeting programmed cell death protein 1 (PD1)/programmed cell death 1 ligand 1 (PD-L1) and cytotoxic T lymphocyte associated protein 4 (CTLA4) co-inhibitory pathway improves overall survival in patients with metastatic tumors.^1^ However, many tumors show resistance to the inhibitors.^2^ Attention has also shifted to activating T cells by stimulating T cell costimulating factors such as OX40, 4-1BB, ICOS, and GITR, members of the tumor necrosis factor receptor superfamily.^3^ Systemic administration of multiple antibodies modulating T cell function may activate T cells that specifically recognize cancer cells and T cells that respond to normal tissue, resulting in several immune-related adverse effects (irAEs).^4^ Local administration of immunotherapy to the tumor may more efficiently activate cancer-specific immunity and reduce the incidence of irAEs.^5^

Hemagglutinating virus of Japan envelope (HVJ-E), a UV-irradiated form of HVJ, shows strong anti-tumor effects although HVJ-E is a non-replicative virus particle.^6^ HVJ-E activates anti-tumor immunity by activating dendritic cells and repression of regulatory T cell (Treg) activity^7^ and eliciting cancer cell death by apoptosis^8^ and necroptosis.^9^ A phase Ia clinical trial of GEN0101 (i.e., clinically applied version of HVJ-E) in patients with advanced malignant melanoma shows local complete or partial responses in 11 of 18 target lesions and a decrease in lung metastases in one patient.^10^^,^^11^ An open-label, phase I, dose-escalation study in patients with castration-resistant prostate cancer (CRPC) elicits stable disease in 1 of 3 patients who received 30,000 milli-neuraminidase units (mNAU) and in 5 of 6 patients who received 60,000 mNAU GEN0101 and reduced metastasis in lymph node in 3 of 6 patients.^12^ These clinical and pre-clinical studies indicate that HVJ-E has substantial anti-tumor effects at the intratumorally injected lesions and moderate systemic anti-tumor effects in 1 of 6 patients with malignant melanoma and 3 of 9 patients with CRPC.

In this study, we demonstrated that combining HVJ-E and anti-OX40 agonist antibody (OX40 antibody) induces systemic anti-tumor effects. HVJ-E administration alone did not significantly increase tumor-infiltrating lymphocytes (TILs). Consistently, PD1 antibody did not promote the anti-tumor activity of HVJ-E both in the target and non-target lesions. Combining Toll-like receptor 9 (TLR9) stimulation with OX40 antibody activates systemic anti-tumor effects.^13^^,^^14^ Although HVJ-E induces the expression of interferon-beta (IFN-β) and IFN-γ independently of the TLR signaling pathway,^15^ combining HVJ-E and OX40 antibody activated CD4 and CD8 T cells at the target lesion. These T cells repress the tumor growth at the non-target lesions. The systemic anti-tumor effects induced by HVJ-E and OX40 antibody required the interaction between Nkg2d ligands (Nkg2d-L) in cancer cells and Nkg2d in T cells. Our findings have the potential to contribute to the development of an anti-tumor therapeutic method based on these mechanisms.

## Results

### HVJ-E + T cell costimulatory molecule-activating antibody induces an abscopal-like effect, increasing tumor-infiltrating T cells at the target and non-target lesions

**Figure 1:**
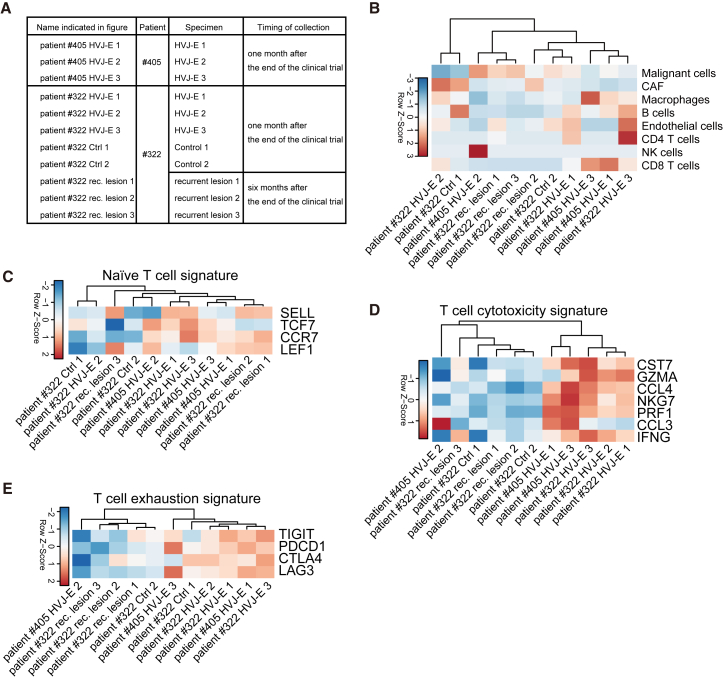
T cell signature gene expression in melanoma specimens from two HVJ-E-treated patients with advanced melanoma (A) Table of collected specimens from the two HVJ-E-treated patients. We obtained three GEN0101 (i.e., clinical application HVJ-E)-treated and two non-treated specimens from patient 322 and three treated specimens from patient 405 one month after GEN0101 administration. From patient 322, we also obtained three distantly located relapsed specimens 6 months after the end of the clinical trial. (B–E) Heatmap of naive T cell signature (B), cytotoxicity signature (C), and exhaustion signature (D), and exhaustion signature (E) gene expression in the bulk RNA-seq data from the indicated melanoma specimens.

Immune cell activation is required for systemic anti-tumor effects. Since HVJ-E can activate CD8 T cells,^7^^,^^16^^,^^17^ we theorized that optimizing the stimulation of immune cells by HVJ-E in tumors will induce systemic anti-tumor effects. We digitally analyzed the proportion of cancer and non-cancer cells in melanoma samples collected from two patients with advanced melanoma who participated in the HVJ-E clinical trial (Figure 1A). GEN0101 (i.e., clinical application HVJ-E) intratumoral injection increased CD8 T cells only in the target lesions (Figure 1B) and increased the expression of genes related to T cell cytotoxicity and exhaustion signatures, but not of those related to naive T cell signature^18^ (Figures 1C–1E). These data suggest that HVJ-E increases the number of CD8 T cells with an activated or exhausted status at the target lesions in patients with advanced melanoma.

**Figure 2:**
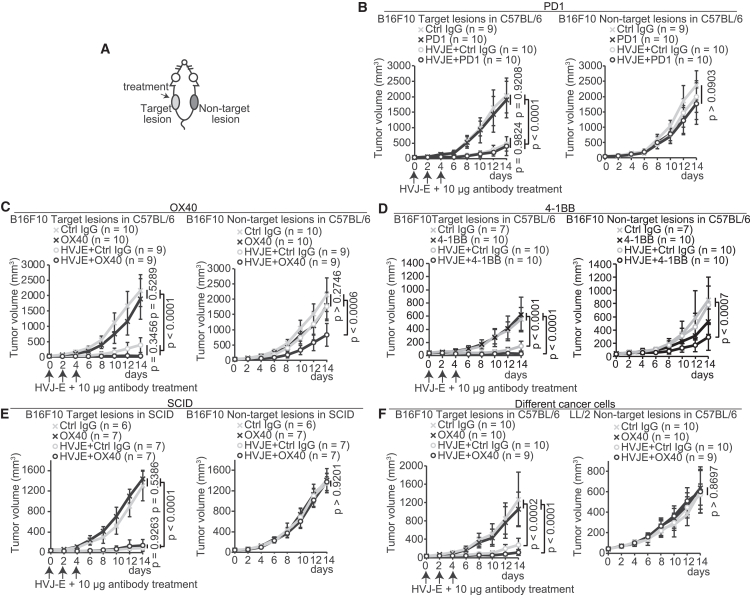
HVJ-E + T cell costimulatory molecule-activating antibody induces an abscopal effect (A) Schema of mouse tumor model bilaterally transplanted with 5 × 10^5^ B16F10 or LL/2 cells. (B–D) The tumor growth curve of B16F10 cells at the target and non-target lesions in C57BL/6N mice. HVJ-E (2,000 HAU) was intratumorally injected with 10 μg anti-PD1 (B), anti-OX40 agonist (C), or anti-4-1BB agonist (D) antibody. (E) The tumor growth curve in SCID mice. (F) The tumor growth curve of B16F10 cells at the target lesion and LL/2 at the non-target legion in C57BL/6N mice. *p* values were calculated using the Turkey HSD test. (B–F) Error bars show the SD.

Immune-modulating antibodies, including anti-PD1 antibody and OX40 antibody, modify immune cell activity in human and mouse tumors.^13^^,^^19^^,^^20^^,^^21^ We thus assessed whether immune-modulating antibodies amplify and promote the migration of locally activated or exhausted T cells in HVJ-E-treated lesions. We generated tumors by bilaterally inoculating mice with B16F10 cells (Figure 2A). Unexpectedly, HVJ-E + anti-PD1 antibody did not suppress tumor growth in the non-target lesion (*p* > 0.0903). However, HVJ-E significantly suppressed tumor growth at the target lesion (*p* < 0.0001, Figure 2B). The data indicate that HVJ-E cannot induce the abscopal-like effect in mouse tumor models, in agreement with the absence of T cell enrichment in the non-target lesions of HVJ-E-treated patients with advanced melanoma.

Next, we assessed whether T cells require an activation signal. HVJ-E + OX40 antibody (HVJ-E/OX40 antibody) significantly suppressed tumor growth at both the target (*p* < 0.0001) and non-target (*p* < 0.0006) lesions, while administration of OX40 antibody alone did not suppress tumor growth in either lesion (*p* = 0.5289 and *p* > 0.2746, respectively; Figure 2C). HVJ-E + anti-4-1BB agonist antibody, another T cell costimulatory molecule known as Tnfrsf9 or Cd137, showed similar tumor growth suppression (Figure 2D). In SCID mice, an immune-deficient mouse line lacking functional T and B lymphocytes, HVJ-E/OX40 antibody did not affect tumor growth at the non-target lesion (*p* > 0.9201, Figure 2E). The systemic anti-tumor effects showed tumor-type specificity because combination therapy at the target lesion of B16F10 cells did not modify tumor growth at the non-target lesion of unmatched mouse Lewis lung carcinoma cell line LL/2 cells (*p* > 0.8697, Figure 2F). The HVJ-E/OX40 antibody also did not decrease body weight (Figure S1A), suggesting that systemic anti-tumor effects are induced without irAEs. These results indicate that the combination of HVJ-E with a T cell costimulatory molecule induces systemic anti-tumor effects dependent on lymphocytes.

**Figure 3:**
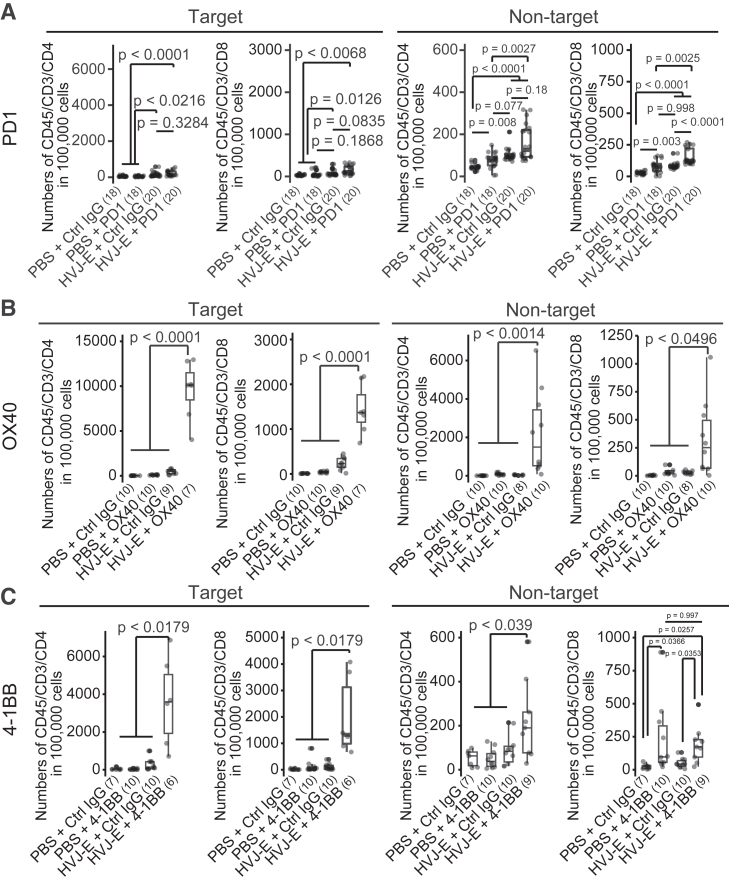
HVJ-E + T cell costimulatory molecule-activating antibody increases tumor-infiltrating T cells at the target and non-target lesions (A–C) Boxplot of CD45/CD3/CD4 and CD45/CD3/CD8 T cell number. HVJ-E (2,000 HAU) was intratumorally injected with 10 μg anti-PD1 (A), anti-OX40 agonist (B), or anti-4-1BB agonist (C) antibody on days 0, 2, and 4. Tumors were analyzed 14 days after treatment initiation. *p* values were calculated using the Steel-Dwass test. Gray dot shows each T cell number. The black dots indicate the outliers. The numbers in parentheses indicate the number of samples.

To examine whether the combination of HVJ-E with immune-modulating antibodies modifies the tumor immune microenvironment, we first analyzed the proportion of tumor-infiltrating T cells using a gating strategy (Figure S1B). Intratumoral injection of HVJ-E alone slightly increased CD4 and CD8 T cells (*p* < 0.0216 and *p* = 0.0126, respectively) at the target lesion, whereas the anti-PD1 antibody alone did not, consistent with previous reports^22^^,^^23^^,^^24^^,^^25^ (Figure 3A). HVJ-E + anti-PD1 antibody also did not significantly increase CD4 and CD8 T cells (*p* = 0.3284 and *p* = 0.0835, respectively) in the target lesion compared with HVJ-E treatment alone. In the non-target lesion, HVJ-E + anti-PD1 antibody moderately increased the proportion of CD8 T cells (*p* < 0.0001) but not CD4 T cells (*p* = 0.18) compared with HVJ-E treatment alone. Consistent with the tumor growth curve data in Figure 2, the HVJ-E/OX40 antibody substantially increased CD4 and CD8 T cells both at the target (*p* < 0.0001) and non-target (*p* < 0.0014 and *p* < 0.0496, respectively) lesions, whereas OX40 antibody alone did not alter the proportion of T cells (Figure 3B). Furthermore, CD4 and CD8 T cells were found activated in tumors with a decreased percentage of PD1-positive cells but no substantial change in that of OX40-positive cells (Figures S1C and S1D). Meanwhile, HVJ-E + anti-4-1BB agonist antibody increased CD4 and CD8 T cells both at the target (*p* < 0.0179) and non-target (*p* < 0.039 and *p* = 0.0257, respectively) lesions, whereas anti-4-1BB agonist antibody alone increased CD8 T cells in the non-target lesion (*p* = 0.0366), which is inconsistent with the absence of increased CD8 T cells at the target lesion (Figure 3C). Considering the adverse effects of the anti-4-1BB agonist antibody in a clinical trial,^26^ the strong stimulation induced may fail to control CD8 T cell activity. Overall, the results indicate that HVJ-E and OX40 antibody synergistically increase tumor-infiltrating T cells.

**Figure 4:**
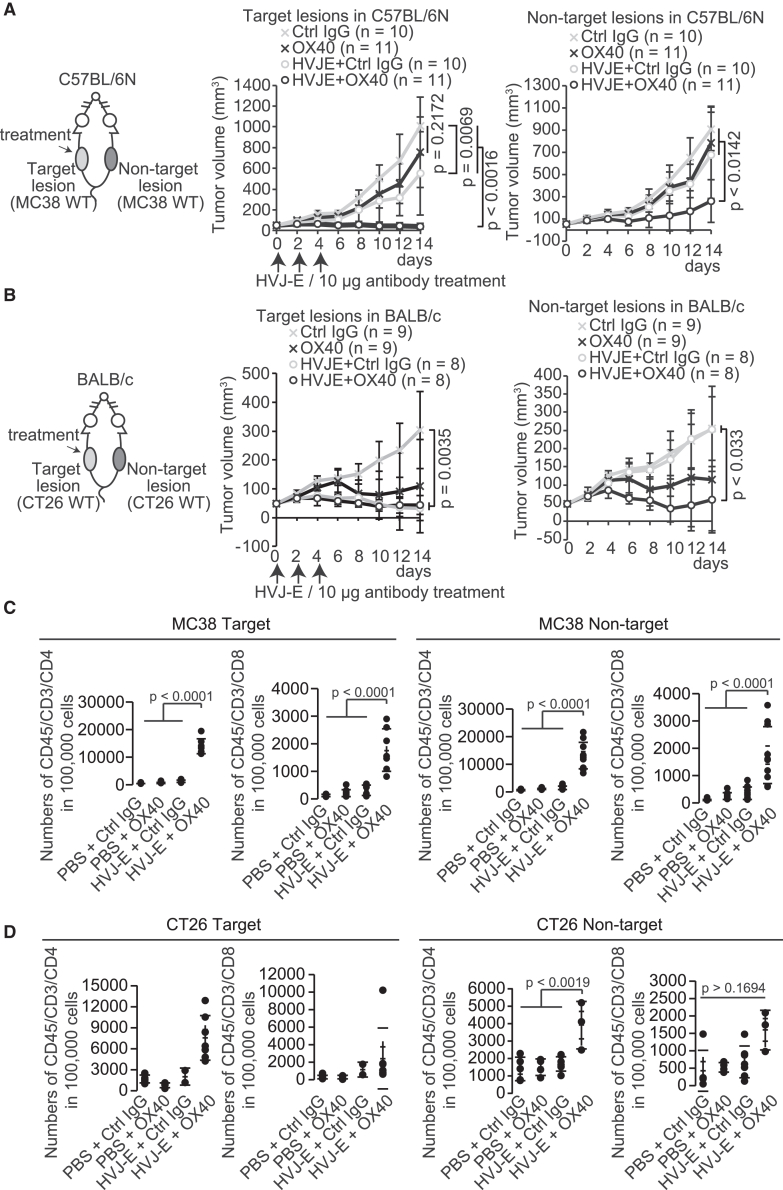
The systemic anti-tumor effects of HVJ-E + anti-OX40 agonist antibody in mice bilaterally inoculated with MC38 and CT26 cells (A and B) Tumor growth curves of MC38 (A) and CT26 (B) cells at the target and non-target lesions in C57BL/6N (A) and BALB/c (B) mice. HVJ-E (2,000 HAU) was intratumorally injected with 10 μg anti-OX40 agonist or Ctrl antibody on days 0, 2, and 4. Error bars show the SD. (C and D) Dot plot of the number of CD45/CD3/CD4 and CD45/CD3/CD8 T cells in 100,000 cells at the target and non-target lesions of MC38 (C) and CT26 (D) cells. (C) PBS + Ctrl antibody, *n* = 8; PBS + anti-OX40 agonist antibody, *n* = 10; HVJ-E + Ctrl antibody, *n* = 9; HVJ-E + anti-OX40 agonist antibody, *n* = 7 for the target lesion. PBS + Ctrl antibody, *n* = 10; PBS + anti-OX40 agonist antibody, *n* = 11; HVJ-E + Ctrl antibody, *n* = 10; HVJ-E + anti-OX40 agonist antibody, *n* = 10 for the non-target lesion. (D) PBS + Ctrl antibody, *n* = 8; PBS + anti-OX40 agonist antibody, *n* = 4; HVJ-E + Ctrl antibody, *n* = 2; HVJ-E + anti-OX40 agonist antibody, *n* = 7 for the target lesion. PBS + Ctrl antibody, *n* = 5; PBS + anti-OX40 agonist antibody, *n* = 4; HVJ-E + Ctrl antibody, *n* = 7; HVJ-E + anti-OX40 agonist antibody, *n* = 3 for the non-target lesion. We did not collect enough cells from the HVJ-E + Ctrl IgG-treated lesions since HVJ-E almost completely eradicated them. *p* values were calculated using the Tukey HSD test and the Steel-Dwass test.

To confirm whether the HVJ-E/OX40 antibody induces systemic anti-tumor effects in different cancer cell types, MC38 and CT26 mouse colon cancer cells were bilaterally inoculated into mice (Figure 4). Consistent with the results of mice bilaterally inoculated with B16F10 cells, the combination therapy significantly suppressed MC38 and CT26 tumor growth in C57BL/6N and BALB/c mice, respectively, at the target (*p* < 0.0016 and *p* = 0.0035) and non-target (*p* < 0.0142 and *p* < 0.033) lesions (Figures 4A and 4B). Moreover, CD4 and CD8 T cell numbers increased at both target (*p* < 0.0001) and non-target (*p* < 0.0001) lesions to a greater extent in the MC38 model than in B16F10 tumors (Figure 4C). Although OX40 antibody alone tended to attenuate CT26 tumor growth at the target and non-target lesions, neither CD4 nor CD8 T cell number increased (Figures 4B and 4D). Moreover, CD8 T cells were not significantly increased in CT26 non-target lesions, suggesting that CT26 cells are vulnerable to the immune system or OX40 antibody. The results suggest that HVJ-E/OX40 antibody therapy is effective against different tumor types.

**Figure 5:**
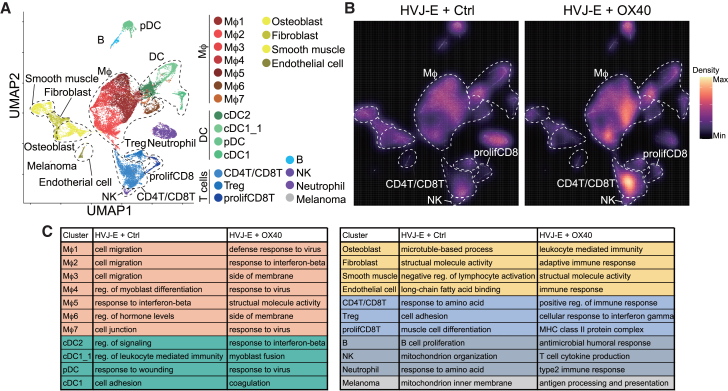
HVJ-E + OX40 agonist antibody modulates tumor microenvironments at the non-target lesions (A) UMAP of 23,156 GFP-positive host cells using single-cell RNA-seq data from the non-target lesion in GFP mice bilaterally inoculated with B16F10 cells. The target lesion was treated with HVJ-E + Ctrl IgG or anti-OX40 agonist antibody. (B) Density plot of the UMAP stratified by treatment. (C) Significantly enriched (*p* < 0.05) gene ontology terms in each cluster detected in (A).

We next investigated the dynamic change in the tumor environment in the non-target lesion by single-cell RNA sequencing (scRNA-seq) of GFP-positive cells from GFP mice bilaterally inoculated with B16F10 cells (Figures 5A and S2A). Consistent with the FACS results, the combination therapy increased CD4 and CD8 T cells as well as macrophages, but not Tregs (Figures 5B and S2B). Compared with the HVJ-E/control antibody treatment, gene set enrichment analysis revealed that HVJ-E/OX40 antibody activated anti-tumor immune responses, such as defense response to virus and response to IFN-β, in most of the clusters (Figure 5C). In addition, scRNA-seq data showed that HVJ-E/OX40 antibody decreased mitochondria-related gene expression and increased the expression of genes related to antigen processing and presentation in melanoma cells (Figure 5C). These data indicate that intratumoral injection of the HVJ-E/OX40 antibody at the target lesion modifies the tumor environment and increases T cells at the non-target lesion, resulting in activation of the anti-tumor immune response.

### HVJ-E + OX40 agonist antibody modulates tumor-infiltrating T cell dynamics via Nkg2d

**Figure 6:**
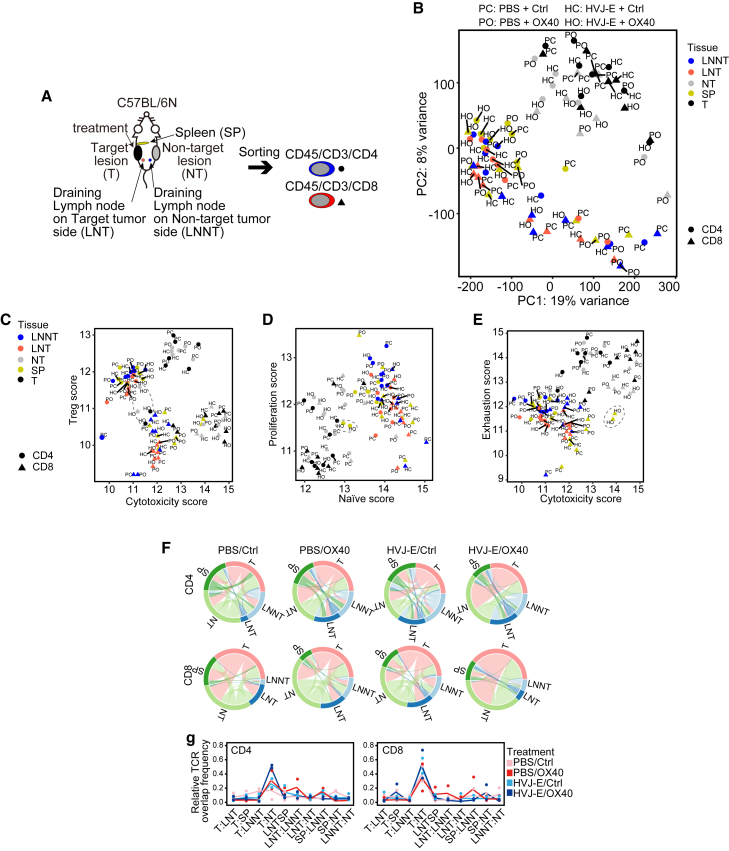
HVJ-E + OX40 agonist antibody modulates tumor-infiltrating T cell dynamics (A) Schema of the treatment and tissue collection from B16F10 bilaterally inoculated mice (*n* = 2 per treatment group). (B) PCA plot of CD4 and CD8 T cell RNA-seq data using the top 5,000 genes. (C–E) Dot plot of T cell status scores calculated from RNA-seq data of CD4 and CD8 T cells. (F) The representative circular plot of TCR β overlap frequency in each mouse. (G) Line plot of the mean of relative overlap frequency between the indicated tissues. The dot indicates each data point.

To elucidate how local administration of the HVJ-E/OX40 antibody activates T cells in distantly located lesions, we assessed CD4 and CD8 T cell status and the traffic between tumors, lymph nodes, and spleen (Figure 6A). We first confirmed the distinct isolation of CD4 and CD8 T cells using T cell marker genes in RNA-seq data (Figure S3A). Tumor-infiltrating T cells showed very different gene expression profiles from those in the lymph nodes and spleen (Figure 6B), consistent with previous reports.^27^^,^^28^ Compared with other control treatments, the HVJ-E/OX40 antibody did not change the overall gene expression profiles (Figure 6B). In the lymph nodes and spleen, most CD4 T cells showed different gene expression profiles from those of CD8 T cells. Tumor-infiltrating CD4 and CD8 T cells were indistinguishable in the overall gene expression profile, but T cells at the target lesion differed from those at the non-target lesion.

Further comparison of the expression of T cell function-related genes showed that CD8 T cells had a high cytotoxicity score, while CD4 T cells had a high Treg score in tumors (Figure 6C). The HVJ-E/OX40 antibody decreased the Treg score of CD4 T cells at the target and non-target lesions, while the proliferation score of CD4 and CD8 T cells at the non-target lesion was higher than at the target lesion (Figure 6D). CD8 T cells at the target lesion showed a higher exhaustion score than those at the non-target lesion (Figure 6E). The HVJ-E/OX40 antibody also decreased the proliferation and naive score of splenic CD4 T cells and increased the cytotoxicity score of splenic CD8 T cells (Figures 6D and 6E). These results suggest that the HVJ-E/OX40 antibody suppresses Treg function in tumor-infiltrating CD4 T cells and activates the splenic CD8 T cells function.

We next detected the HVJ RNA genome in the RNA-seq data of T cells from HVJ-E-administered mice to examine whether T cells migrate from the target lesion to other tissues. Although the number of reads assigned to the HVJ genome was small, we consistently detected the HVJ genome in CD4 and CD8 T cells at the target and non-target lesions in two mice administered HVJ-E/OX40 antibody, suggesting T cell migration from the target to the non-target lesions (Figure S3B). The infectivity of HVJ-E to T cells was confirmed by FACS (Figure S3C). To further test this, we utilized the T cell receptor beta (TCR-β) repertoire data, which cover the vast majority of TCRs, as most CD4 and CD8 T cells expressed TRA and TRB chains (Figures S4A and S4B). We detected an overlap of TCR β between tissues (Figure 6F), and each mouse had a different TCR β repertoire even after receiving the same treatment (Figure S4C). CD4 T cells showed an increased overlap frequency between the target and non-target lesions after HVJ-E/OX40 antibody injection, whereas overlap frequencies of CD8 T cells were similar among all treatment groups despite the increase in CD8 T cell number after HVJ-E/OX40 antibody injection (Figure 6G). The signal pathway and cell-cell communication analysis in scRNA-seq of the non-target tumors in HVJ-E/OX40 or HVJ-E/Ctrl antibody-treated mice suggested that the HVJ-E/OX40 antibody enhances the tumor necrosis factor signal pathway and communication between a population of macrophage and proliferative CD8 T cell (Figures S5A–S5C). These results suggest that T cells are trafficked between tumors and proliferation of the T cells at tumors.

**Figure 7:**
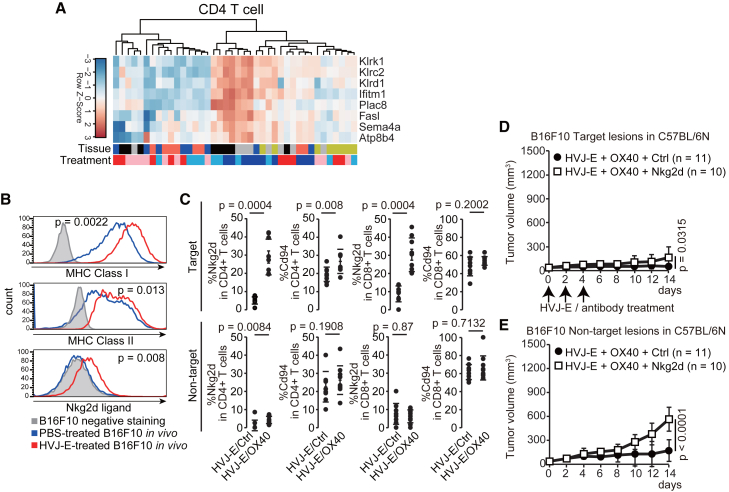
The association between Nkg2d-L and Nkg2d is critical for modulating tumor-infiltrating T cell dynamics by HVJ-E + OX40 agonist antibody (A) Heatmap of Klrk1- or Fasl-containing cluster in CD4 T cells. (B) MHC class I, MHC class II, Nkg2d ligand expression in B16F10 cells *in vivo*. PBS, *n* = 9; HVJ-E, *n* = 10. (C) Percentage of Nkg2d and Cd94 expression in CD45/CD3/CD4 and CD45/CD3/CD8 T cells; HVJ-E (2,000 HAU) was intratumorally injected with 10 μg anti-OX40 agonist antibody on days 0, 2, and 4. Tumors were analyzed 14 days after treatment initiation. HVJ-E/Ctrl antibody-administrated target tumor, *n* = 9; HVJ-E/OX40 antibody-administrated target tumor, *n* = 9; HVJ-E/Ctrl antibody-administrated non-target tumor, *n* = 9; HVJ-E/OX40 antibody-administrated non-target tumor, *n* = 10. (D and E) The tumor growth curve of B16F10 cells at the target (D) and non-target lesions (E) in C57BL/6N mice. Anti-Nkg2d or control antibody at 250 μg in 200 μL was intraperitoneally injected on days −1, 0, 2, 4, and 6. Error bars show the SD. The numbers in parentheses indicate the number of samples. *p* values were calculated using the Wilcoxon test (B and C), Welch’s t test (D), and t test (E), according to the normality and variance of the data.

To identify which genes are involved in the T cell-dependent anti-tumor effect in tumors, we performed gene ontology (GO) enrichment analysis of CD4 and CD8 T cells between each treatment. Consistent with the principal-component analysis of gene expression in T cells, GO enrichment data did not explain the anti-tumor effects at the non-target lesions (Figures 6B and S6). Comparing T cell gene expression between treatments revealed a significant increase in *Klrk1*, *Klrc2*, *Klrd1*, *Fasl*, and *Sema4a* in CD4 T cells at the tumor site after HVJ-E/OX40 antibody injection (Figures 7A and S7). Nkg2d, encoded by *Klrk1*, binds to Nkg2d ligands (NKG2D-L) and acts as a costimulatory molecule for cytotoxic T cells and NK cells.^29^ Cd94, encoded by *Klrd1*, recognizes MHC class I molecules with Nkg2c, encoded by *Klrc2*, and acts as an activating receptor.^30^ CD4 T cells usually do not express Nkg2d; however, a large proportion of tumor-infiltrating NKG2D+ CD4 T cells that release FAS ligands (FASL) are present in patients with tumors.^31^ Correspondingly, HVJ-E increased MHC class I (*p* = 0.0022), MHC class II (*p* = 0.013), and NKG2D-L (*p* = 0.008) expression on the B16F10 cell surface *in vivo* (Figure 7B). Thus, we analyzed Nkg2d and Cd94 protein expression on the T cell surface. Compared with the HVJ-E/control antibody, the HVJ-E/OX40 antibody increased the proportion of Nkg2d+ CD4 (*p* = 0.0004), Cd94+ CD4 (*p* = 0.008), and Nkg2d+ CD8 (*p* = 0.0004) T cells but not that of Cd94+ CD8 T cells (*p* = 0.2002) at the target lesion (Figure 6C). There was no significant increase in Nkg2d and Cd94 in T cells at the non-target lesion, although we detected a significant difference in Nkg2d+ CD4 T cells between HVJ-E/control and HVJ-E/OX40 antibodies. We then assessed whether Nkg2d mediates the HVJ-E/OX40 antibody-induced systemic anti-tumor effects. Nkg2d neutralizing antibody^32^ inhibited the suppression of both target and non-target tumor growth induced by the HVJ-E/OX40 antibody (target, *p* = 0.0315; non-target, *p* < 0.0001; Figures 7D and 7E). The HVJ-E/OX40 antibody also stimulated Nkg2d expression in CD4 and CD8 T cells at the target lesion in mice bilaterally inoculated with MC38 cells (Figure S8). These results suggest a pivotal role of Nkg2d in T cell recognition of cancer cells at the target lesion that activates T cells and suppresses tumor growth at the non-target lesion.

**Figure 8:**
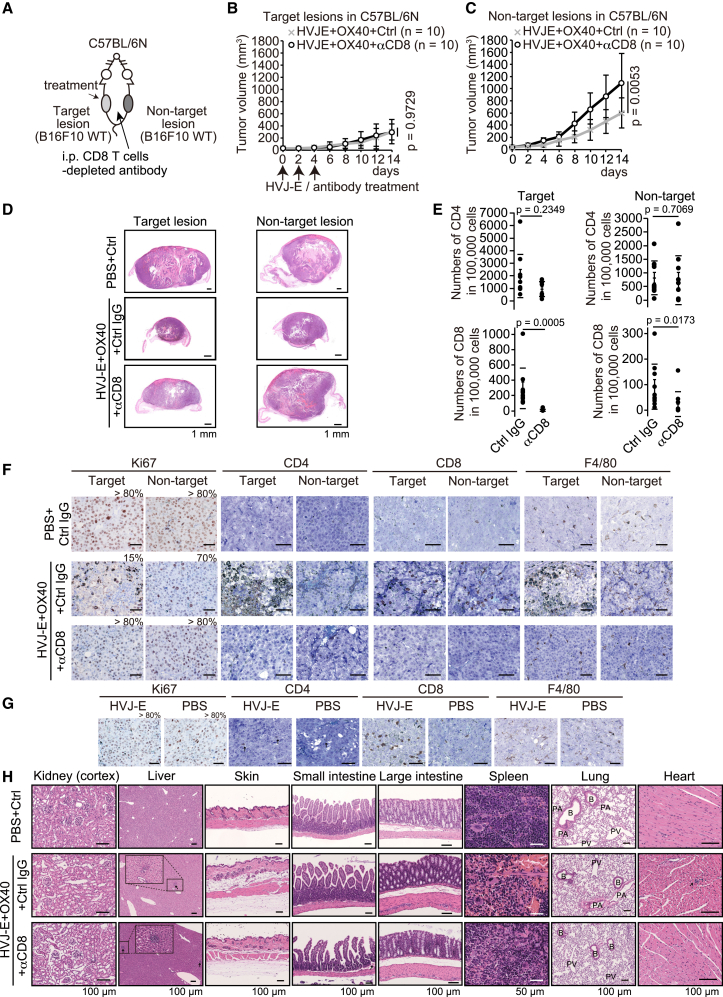
HVJ-E + OX40 agonist antibody-induced anti-tumor effects require CD8 T cells (A) Schema of the mouse tumor model bilaterally transplanted with 5 × 10^5^ B16F10 cells. (B and C) Tumor growth curve of B16F10 cells at the target (B) and non-target (C) lesions in C57BL/6N mice. HVJ-E (2,000 HAU) was intratumorally injected with 10 μg anti-OX40 agonist antibody on days 0, 2, and 4. Anti-CD8 (100 μg) T cell-depleted antibody and 500 μg control antibody in 200 μL PBS were intraperitoneally injected on days −1, 0, 2, 4, and 6. Error bars show the SD. (D) Hematoxylin and eosin staining of each sectioned tissue from B16F10 bilaterally inoculated mice treated with PBS/Ctrl antibody, HVJ-E/OX40 antibody + Ctrl IgG or + anti-CD8 antibody. Scale bar, 1 mm. (E) Dot plot of the number of CD45/CD3/CD4 and CD45/CD3/CD8 T cells in 100,000 cells at the target and non-target lesions. Mice were treated with HVJ-E/OX40 antibody + Ctrl IgG (*n* = 10) or + anti-CD8 antibody (*n* = 10). (F) Immunohistochemistry of each sectioned tissue from B16F10 bilaterally inoculated mice treated with PBS/Ctrl antibody, HVJ-E/OX40 antibody + Ctrl IgG, or + anti-CD8 antibody. The indicated antibodies were used for staining. The percentage indicates the Ki67 labeling index. Representative images are from two or three mice. Scale bar, 50 μm. (G) Immunohistochemical staining of each sectioned tissue from B16F10 inoculated mice treated with HVJ-E or PBS. The indicated antibodies were used for staining. The percentage indicates the MIB-1 index. Scale bar, 50 μm. (H) Representative images are from three mice. In the liver and heart panels, arrows indicate small foci of lymphocyte aggregates with fewer than 100 lymphocytes. In the intestine panels, arrows indicate vasodilation with congestion. In the lung panel, B, bronchus or bronchiole; PA, pulmonary artery; PV, pulmonary vein. (B, C, and E) *p* values were calculated using a t test.

Next, to examine whether CD8 T cells are critical for the suppression of tumor growth at the non-target lesion, we immune-depleted CD8 T cells in HVJ-E/OX40 antibody-treated mice (Figure 8A). CD8 T cell depletion showed comparable tumor suppression at the target lesion to the control (*p* = 0.9729, Figures 8B, 8D, and 8E). At the non-target lesion, CD8 T cell depletion attenuated the HVJ-E/OX40 antibody-induced tumor suppression (*p* = 0.0053, Figures 8C–8E). The Ki67 tumor-cell proliferative activity was decreased in HVJ-E/OX40 + Ctrl antibody- but not + anti-CD8 antibody-treated mice (Figures 8F and 8G). CD4 and F4/80-positive cells were histologically increased at the target lesion in HVJ-E/OX40 + Ctrl antibody- but not in HVJ-E/OX40 + anti-CD8 antibody-treated mice (Figures 8F and 8G). While a few scattered foci of lymphocyte aggregates were observed in the liver and heart of all HVJ-E/OX40 antibody treatment groups, we did not find any severe inflammation in HVJ-E/OX40 antibody-treated mice, suggesting that HVJ-E/OX40 antibody treatment does not cause irAEs (Figure 8H). These data indicate that CD8 T cells have a critical role in HVJ-E/OX40 antibody-induced systemic anti-tumor effects.

## Discussion

The combination of HVJ-E with a T cell costimulatory molecule stimulation activated T cells and repressed the tumor growth at the target and the non-target lesions. In the HVJ-E/OX40 antibody-treated mice, we observed T cell migration from the target to the non-target lesion, but there was little overlap of TCR between the tumor and draining lymph nodes. Our findings suggest a substantial correlation between T cells among tumors in mice with systemic anti-tumor effects. The local administration of HVJ-E/OX40 antibody has the potential to serve as a therapeutic approach for inducing systemic anti-tumor effects.

The local virotherapy treatment, including HVJ-E, represses tumor growth at the non-target lesion in certain patients, while its efficacy varies among individuals.^11^^,^^33^ Our analysis of gene expression profile in the target and non-target tumors in patients who received HVJ-E administration detected the activated T cells in the target lesions 1 month after administration and a limited increase of T cells in the non-target lesions. Consistent with the findings in humans, HVJ-E administration alone showed significant anti-tumor effects in the target lesion in mouse models. However, the response in the non-target lesion was limited. A previous report indicates that HVJ-E administration alone activates anti-tumor immunity, and the anti-tumor effects of HVJ-E are attenuated in immunocompromised mice even in the target lesion^7^; however, we found that HVJ-E represses tumor growth at the target lesion independently of the immune system using several cancer cell lines. This disparity might be attributed to contamination, such as mycoplasma, in the cancer cells. These findings suggest that the anti-tumor effects of HVJ-E at the target lesion are independent of T, B, and NK cells and the limited effects of HVJ-E at the non-target lesion, at least in mouse models.

Combining HVJ-E with PD1 antibody did not activate systemic anti-tumor effects. HVJ-E administration alone did not activate and proliferate T cells at the target lesions, suggesting that HVJ-E alone did not convert tumor status from cold to hot. Thus, blocking the immune checkpoint could not activate T cells in HVJ-E-treated tumors. In humans, HVJ-E treatment increased the expression of active T cell-related genes at the target lesions. Some patients show a response to HVJ-E at the non-target lesion.^10^^,^^11^ These data suggest that humans may be more sensitive to HVJ-E treatment than mouse tumor models. Other virotherapies, such as adenovirus, herpes simplex virus, and vaccinia virus, followed by immune checkpoint inhibitors, show durable response rates in subsets of patients.^33^ However, combining herpes simplex virus-based talimogene laherparepvec with pembrolizumab or ipilimumab did not significantly increase overall survival and progression-free survival in patients with advanced-stage immunotherapy-naive melanoma compared with antibody alone.^34^^,^^35^ These data suggest that combining virotherapy with immune-activating antibodies, including OX40 agonist antibody, could yield significant anti-tumor effects.

The local treatment combining HVJ-E with OX40 or 4-1BB antibody repressed the tumor growth at the target and non-target lesions. In contrast, these antibodies alone did not show significant anti-tumor effects. The local treatment combining TLR9 agonist with OX40 antibody activates systemic anti-tumor effects.^13^ The combination therapy requires the collaboration between T and B cells.^36^ While HVJ-E triggers RIG-I signaling pathway,^8^ HVJ-E is independent of the TLR signaling pathway.^15^ The detailed molecular mechanisms of how OX40 antibody acts with RIG-I and TLR9 stimulation to activate T cells are unclear. However, we found that the association of Nkg2d-L in cancer cells with Nkg2d in T cells is pivotal for the systemic anti-tumor effects of HVJ-E and OX40 antibody combination therapy. This finding suggests that promoting the association of cancer cells with T cells serves as a trigger to elicit systemic anti-tumor effects. Subsequently, T cell costimulation may facilitate the activation of T cells. Although further studies are required to elucidate the mechanisms of how the combination therapy activates anti-tumor immunity, our findings suggest that HVJ-E with T cell costimulating molecule stimulation activates systemic anti-tumor effects.

### Limitation

Combining HVJ-E with 4-1BB antibody activated systemic anti-tumor effects similar to those observed with the OX40 antibody. However, 4-1BB antibody alone unexpectedly led to an increase in T cells at the non-target lesions without a corresponding increase at the target lesions. It is still uncertain whether the unexpected effects on T cells are attributed to the properties of the antibody or to 4-1BB itself. Recently, a study suggested that lymph node resection does not affect the efficacy of immune checkpoint inhibitors.^37^ This report may be consistent with our finding of little overlap of TCR between the tumor and draining lymph nodes. Future studies will reveal how the local T cells regulate the tumor growth at the non-target lesions.

## Materials and methods

### Clinical specimens from patients participating in the clinical trial of HVJ-E

Tumor lesions were surgically excised from two patients who participated in the clinical trial of HVJ-E and received a high dose (60,000 mNAU) of HVJ-E. The experiments were approved by the Osaka University Ethics Committee (approval numbers 709 and 15019-2). We collected three tumors and two non-target tumors from patient 322 approximately 1 month following the last HVJ-E treatment. Five months after the final HVJ-E treatment, three independent distant recurrent tumors were surgically excised from patient 322. Three tumors were surgically excised from patient 405 about 1 month following the last HVJ-E treatment. RNA was extracted using ISOGEN (311-02501, Nippon Gene) and ethachinmate (312-01791, Nippon Gene) after homogenizing the tissues with a Beads Cell Disrupter (Yasui Kikai).

### Cell lines and cell culture

Mouse B16F10 melanoma cells (CRL-6475), LL/2 Lewis lung carcinoma cells (CRL-1642), and CT26 colon carcinoma cells (CRL-2638) were purchased from the American Type Culture Collection. Mouse MC38 colon carcinoma cells (ENH204) were purchased from Kerafast. B16F10, LL/2, and MC38 were cultured in DMEM medium (08458-45, Nacalai Tesque) containing 10% FBS (172012, Sigma), 100 U/mL penicillin, and 100 μg/mL streptomycin (26253-84, Nacalai Tesque). CT26 was cultured in RPMI 1640 (30264-56, Nacalai Tesque) medium containing 10% FBS, 100 U/mL penicillin, and 100 μg/mL streptomycin. The cells were cultured at 37°C in a humidified atmosphere of 95% and 5% CO_2_.

### HVJ-E production

The production of HVJ (VR-105 parainfluenza1 Sendai/52, Z strain)-E using virus-free chicken eggs and UV irradiation was previously described.^7^ HVJ-E was verified to be free from mycoplasma- and endotoxin-contamination (6601, TaKaRa and 296-81501, FUJIFILM Wako).

### Tumor mouse model

All mouse experiments were approved by the Osaka University Animal Experiments Committee and were performed under the guidelines. The experimental endpoint of tumor growth was determined as reaching a tumor diameter of 2 cm in any direction, measured using a digital caliper. All cancer cells were verified to be free from mycoplasma contamination through PCR analysis (6601, TaKaRa). B16F10, MC38, or LL/2 cancer cells (0.5 × 10^6^) in 50 μL PBS were intradermally injected into 6- to 8-week-old female C57BL/6N mice. CT26 cells (0.5 × 10^6^) were intradermally injected into 6- to 8 week-old female BALB/cA mice. Mice were maintained under specific pathogen-free conditions. C57BL/6N mice were purchased from Charles River. CB17/IcrJcl-*Prkdc*^*scid*^ (SCID) and BALB/cAJcl mice were purchased from CLEA Japan. HVJ-E treatment commenced when the inoculated tumors reached a diameter of 4.5–5.5 mm, typically 4–6 days post-inoculation. HVJ-E (2,000 hemagglutination units [HAU]) was intratumorally administrated three times every other day. For the mouse model with bilateral tumors, 2,000 HAU HVJ-E with 10 μg of OX40 agonist antibody (Ultra-LEAF Purified anti-mouse CD134 [OX-40] antibody, OX86, 119431, BioLegend), 4-1BB agonist antibody (BE0169, BioXCell), or control antibody (IgG from Rat Serum, 14131, Sigma-Aldrich) were intratumorally injected into the left side of the tumor three times every other day. Tumor size was assessed every other day, with volumes calculated using the formula: tumor volume (mm^3^) = length × (width)^2^/2.

### Flow cytometry analysis and cell sorting

The harvested tumor tissues were finely minced with scissors and then incubated in 2 mL of 0.5% collagenase and 2% fetal bovine serum (FBS) in PBS pre-warmed at 37°C. The minced tissues were incubated for 45–60 min, with pipetting every 15 min at 37°C. The spleen was ground on a 40-μm cell strainer (352340, Falcon) with a syringe plunger in 2–5 mL of 2% FBS/PBS. The dispersed cells were diluted with 8 mL of 2% FBS/PBS and then passed through a 70-μm cell strainer (352350, Falcon). Subsequently, the cell strainer was rinsed with 10 mL of 2% FBS/PBS. After centrifugation of the cells at 1,500 rpm at 4°C for 5 min, the resulting cell pellets were treated with 1–2 mL hemolysis buffer (0.17 M NH_4_Cl, 0.01 M KHCO_3_, 0.082 mM EDTA [pH 7.3]) for just 5 min with gentle shaking. The hemolysis reaction was halted by adding a 10-fold volume of 2% FBS/PBS. Finally, the hemolysis-treated cells were filtered using a 40-μm cell strainer (352340, Falcon).

The dispersed cells were stained with fluorescent-labeled antibodies in 50 μL of 2% FBS in PBS for 30 min on ice. The stained cells were fixed in 4% PFA in PBS after washing twice with 2% FBS in PBS and centrifuging at 600 × *g* and 4°C for 3 min. The intracellular proteins were stained using 1× permeabilization buffer (2106783, Invitrogen) following the manufacturer’s instructions. To analyze T cell function, T cells were stimulated for 6 h with 20 ng/mL PMA (162–23591, FUJIFILM) and 2 μg/mL ionomycin (095-05831, FUJIFILM) in the presence of 20 μg/mL Brefeldin A (022–15991, FUJIFILM) as the Golgi plug. All samples were passed through a Cell-Strainer Cap (352235, Falcon) and analyzed using a FACS Canto2 (BD). For cell sorting, FACS Aria II and FACS Aria IIIu (BD Biosciences) were utilized after staining the cells as described above without fixation.

### RNA-seq and scRNA-seq

Sequencing libraries were prepared from at least two biological replicate RNA samples using an NEBNext Poly(A) mRNA Magnetic Isolation Module (no. E7490, New England Biolabs) and NEBNext Ultra RNA Library Prep Kits for Illumina (no. E7530, New England Biolabs) as described previously.^38^ The prepared sequencing libraries were then analyzed using a HiSeq X instrument (Illumina).

Total RNA was isolated from the isolated CD4 and CD8 T cells using ISOGEN and ethachinmate following the manufacturer’s instructions as described previously.^38^ Sequencing libraries from the two biological replicates of isolated CD4 and CD8 T cells were constructed using the NEBNext Single Cell/Low Input RNA Library Prep Kit for Illumina (E6420L, New England Biolabs) following the manufacturer’s instructions as described previously.^38^ Two biological replicates of scRNA-seq libraries were constructed from the live cells isolated from the non-target lesions of GFP mice bilaterally inoculated with B16F10 cells using the Chromium Controller and Chromium Single Cell 3′ GEM, Library & Gel Bead Kit v.3 (PN-1000092, 10X Genomics). The host-derived live cells were isolated as GFP-positive and DAPI-negative using FACS Aria. Sequencing libraries were analyzed using HiSeq X (Illumina).

### TCR-seq

First-strand cDNA was generated from the total RNA isolated from the isolated CD4 and CD8 T cells using Maxima H Minus Reverse Transcriptase (EP0751, Thermo Fisher Scientific), AS oligo: 5′-AAGCAGTGGTATCAACGCAGAGTACTTTTTTTTTTTTTTTTTTTTTTTTTTTTTTVN, and template switch oligo (TSO): 5′-AAGCAGTGGTATCAACGCAGAGTGAATrGrGrG or NEBNext Single Cell/Low Input RNA Library Prep Kit for Illumina (E6420L, New England Biolabs). The RNA was denatured at 65°C for 5 min in a thermal cycler with a lid temperature of 70°C. The denatured RNA was immediately incubated on ice. The RNA was mixed with 5× RT buffer, 20 U RNase inhibitor, and 100 U Maxima H Minus RT in a total volume of 20 μL. The mixed sample was incubated at 50°C for 30 min in a thermal cycler and then deactivated at 85°C for 5 min. The first PCR amplified the fragment from the TSO to TRBC sequence using Q5 High-Fidelity DNA polymerase (no. 0491L, New England Biolabs), Q5 Reaction Buffer X 5 (no. B9027, New England Biolabs), 5% DMSO, TSO-library-S: ACACTCTTTCCCTACACGACGCTCTTCCGATCTAAGCAGTGGTATCAACGCAGAGT, and mTRBC-library-AS: GACTGGAGTTCAGACGTGTGCTCTTCCGATCTGGAGACCTTGGGTGGAGTCA with a temperature setting of 98°C for 30 s, 30 cycles of 98°C for 10 s, 72°C for 30 s, and 72°C for 2 min. PCR products with a length of approximately 600 bp were collected using AMPure XP (A63881, Beckman Coulter) following the manufacturer’s instructions. The ratio of AMPure XP to PCR sample was 0.55:1. The second PCR amplified the fragment including the CDR3 region and attached sequencing adaptors using 500 pg of the first PCR fragment, NEBNext Q5 Hot Start HiFi PCR Master Mix 2X (E6625AA, New England Biolabs), PE1.0: AATGATACGGCGACCACCGAGATCTACACTCTTTCCCTACACGACGCTCTTCCGATCT, and iPCRtag_X_L2: CAAGCAGAAGACGGCATACGAGATNNNNNNGTGACTGGAGTTCAGACGTGTGCTCTTCCGATCT with a temperature setting of 98°C for 30 s, 12 cycles of 98°C for 10 s, 65°C for 75 s, and 65°C for 5 min. PCR products with a length of approximately 600 bp were collected using AMPure XP from two or three reactions of the second PCR. The ratio of AMPure XP to PCR sample was 0.65:1. PCR product lengths were analyzed using TapeStation (Agilent Technologies). Sequencing libraries were analyzed using HiSeq X (Illumina).

### Infectivity of HVJ-E to T cell

T cells were isolated from the spleen using the EasySep Mouse T cell isolation Kit (no. 19851, STEMCELL Technologies). HAU HVJ-E (10,000) or trypsinized HVJ-E was resuspended in 200 μL Dilutant C and mixed with 8 μL PKH in 200 μL Dilutant C to label HVJ-E with PKH (PKH26GL-1KT, Sigma). After 2–5 min incubation, the reaction was stopped by adding 400 μL 10% FBS RPMI 1640 medium. The PKH-labeled HVJ-E was washed with PBS three times. The PKH-labeled HVJ-E was incubated with 1 × 10^5^ isolated T cells in a 6-well plate for 6 h at 37°C in 5% CO_2_, then analyzed by FACS.

### Histopathological analysis

Tissues were collected from HVJ-E/OX40 antibody/anti-CD8- or control IgG antibody-treated mice (*n* = 3). Tumor tissues were collected from mice 2 weeks after the first treatment. The collected tissues were fixed in 10% neutral buffered formalin solution (Muto Pure Chemicals). After several hours, the tissues were transferred to a new neutral buffered formalin solution and incubated for ∼48 h. Paraffin embedding and sectioning were performed according to a general protocol. Sectioned tissues on glass slides were stained with H&E. Immunohistochemical analysis was performed using antibodies shown in Table S1, as described previously.^39^

### Statistics

We assessed data normality using the Shapiro-Wilk test and verified equal variance between two samples using a two-tailed t test. Parametric data were analyzed using Student’s two-tailed t test, while Welch’s t test was employed for normally distributed, heteroscedastic data. Non-parametric data were analyzed using the Wilcoxon rank-sum test for comparing two samples and one-way ANOVA with Tukey’s HSD test for comparing multiple groups. Steel tests were applied for comparing multiple nonparametric samples. Error bars represent the standard deviation. Calculations were performed using JMP Pro 13 software.

### Bioinformatics

#### Software used in the study

bedtools v.2.26.0^40^

bowtie2 2.2.3^41^

Cell Ranger 3.1.0 (10× GENOMICS).

CellChat 1.1.3^42^

CIBERSORTx (https://cibersortx.stanford.edu/)^43^

clusterProfiler R package 3.14.3^44^

DESeq2 1.26.0^45^

DOSE 3.12.0^46^

enrichplot R package 1.6.1 (https://yulab-smu.top/biomedical-knowledge-mining-book/).

FastQC v.0.11.5 (https://www.bioinformatics.babraham.ac.uk/projects/fastqc/).

ggplot2 R package 3.3.3 (http://ggplot2.org).^47^

ggrepel R package 0.9.1 (https://cran.r-project.org/web/packages/ggrepel/index.html).

gplots R package 3.1.1 (https://cran.r-project.org/web/packages/gplots/index.html).

IGV 2.3.91^48^^,^^49^

IGVtools 2.3.91^49^

MIXCR v.3.0.7^50^

org.Mm.eg.db 3.10.0 (https://bioconductor.org/packages/release/data/annotation/html/org.Mm.eg.db.html).

R 3.6.3 (https://www.r-project.org).

Rstudio 1.0.44 (https://www.rstudio.com).

Rstudio Server v.1.4.1103 (https://www.rstudio.com/products/rstudio/download-server/).

Samtools 0.1.17^51^

Seurat 3.2.2^52^

STAR 2.5.3a^53^

Stringtie v.1.3.4b^54^

VDJtools 1.2.1^55^

#### RNA-seq data analysis

The STAR aligner mapped paired-end reads to the mouse reference genome mm9 after checking the read quality using FastQC. Stringtie and DESeq2 calculated gene expression levels. Gene expression correlation between replicates was computed using R in Table S2. IGVtools converted the wig files to tdf format to create sequencing tracks, using the option -z 7 and the tracks were visualized using IGV, as described previously.^38^ A gene expression heatmap was created using heatmap.2 in the gplots R package. PCA plots were generated using DESeq2. GSEA was computed, using clusterProfiler, enrichplot, DOSE, and org.Mm.eg.db. Biological process was utilized as the GO term.

T cell status scores were defined as the average relative gene expression of each gene set for cytotoxicity: Nkg7, Ccl4, Cst7, Prf1, Gzma, Gzmb, Ifng, and Ccl3; exhaustion: Pdcd1, Tigit, Lag3, Havcr2, Ctla4; Naive, Ccr7, Tcf7, Lef1, and Sell; and proliferation: Mki67, Hist1h1d, Pcna, Smc4, Mcm3; Treg, Batf, Foxp3, Ikzf2, Arid5b, Prdm1, Vdr, and Maf, as described previously.^18^ The proportion of TRA, TRB, TRD, and TRG chains was analyzed using MIXCR^50^ with the option shotgun.

#### scRNA-seq data analysis

BCL files of paired-end reads were analyzed using Cell Ranger to obtain the gene expression count matrix. The obtained data were merged and analyzed using Seurat. Cell-cell communication between clusters was analyzed using CellChat.

#### Digital cytometry analysis

Relative gene expression values in the clinical specimens were analyzed to calculate the proportion of tumor comprising cells using CIBERSORTx.^43^

#### HVJ-E RNA detection

HVJ-derived RNA was detected from RNA-seq data. Paired-end reads were mapped to the mouse reference genome mm9 and HVJ complete genome M30202 by STAR using the following options: --outSAMattributes NH HI AS nM NM XS, --twopassMode Basic, --outFilterMatchNmin 3, --outFilterScoreMinOverLread 0.6, --outFilterMatchNminOverLread 0.6, as described previously for viral track scanning.^56^

#### TCR-seq data analysis

Paired-end reads of TCR-seq libraries were analyzed using MIXCR^50^ with the following parameters: amplicon, --starting-material rna, --5-end no-v-primers, --3-end c-primers, and --adapters adapters-present. The obtained TRB data were processed to eliminate erroneous clonotypes and non-functional clonotypes, normalized to 3,000, and plotted using VDJtools.^55^ The dependencies between sample diversity and sample size were calculated using the option of rarefaction in VDJtools.

## Data and code availability

Sequencing data were deposited to PRJDB11300 and PRJDB11390 in DNA Data Bank of Japan (DDBJ).

## Acknowledgments

We thank Mayuko Okado, Koki Oyama, Hayato Mori, Kenji Oyachi, and Junko Kumagai for technical assistance; Dr. Yohei Mikami for critical reading, and Dr. Mariko Okada and Dr. Keita Iida for providing technical advice. A.I. was supported by Research Fellow of 10.13039/501100001691Japan Society for the Promotion of Science (JP22J15287). This work was supported by the Center for Medical Research and Education, Graduate School of Medicine, 10.13039/501100004206Osaka University; 10.13039/100009619AMED grant nos. 18cm0106341h0001 and 23ym0126809j0002; 10.13039/501100001691JSPS 10.13039/501100001691KAKENHI grant nos. JP21K19408, JP21H05158, and JP21H05160; research grants from Bristol-Myers Squibb, The Osaka Community Foundation, and Osaka University Entrepreneurship Development Grant; and in part by the Osaka University Program for the Support of Networking among Present and Future Researchers (to K.N.).

## Author contributions

A.I. and K.N. designed the experiments. A.I., Y.L., , Y.U., K.K., K.S., Y.Y., S.I., K.I., S.I., and K.N. performed the experiments. R.O. performed histological analyses. K.N. performed bioinformatic analysis. A.T. and E.K. collected the clinical specimens. S.H., H.Y., and Y.K. provided expert advice. K.N. designed and supervised the study and wrote the manuscript.

## Declaration of interests

A.I. and K.N. applied for a patent based on the study with Osaka University.

## Supplemental information

Document S1. Figures S1–S8 and Tables S1–S3

Document S2. Article plus supplemental information
