# Supplementary material for: Local treatment of HVJ-E with T cell costimulatory molecule stimulation elicits systemic anti-tumor effects
Source: Mol Ther Oncol. 2024 Oct 10;32(4):200893. doi: 10.1016/j.omton.2024.200893 (PMC11555341; doi:10.1016/j.omton.2024.200893)
Supplement: Document S1. Figures S1–S8 and Tables S1–S3 [file mmc1.pdf]

OMTON, Volume *32*

Supplemental information

Local treatment of HVJ-E with T cell costimulatory molecule stimulation elicits systemic anti-tumor effects

Airi Ishibashi, Yue Li, Yuuta Hisatomi, Noriko Ohta, Yuko Uegaki, Atsushi Tanemura, Riuko Ohashi, Koji Kitamura, Kotaro Saga, Yasuhide Yoshimura, Satoko Inubushi, Kyoso Ishida, Sadahiro Iwabuchi, Shinichi Hashimoto, Eiji Kiyohara, Hideo Yagita, Yasufumi Kaneda, and Keisuke Nimura

Figure S1

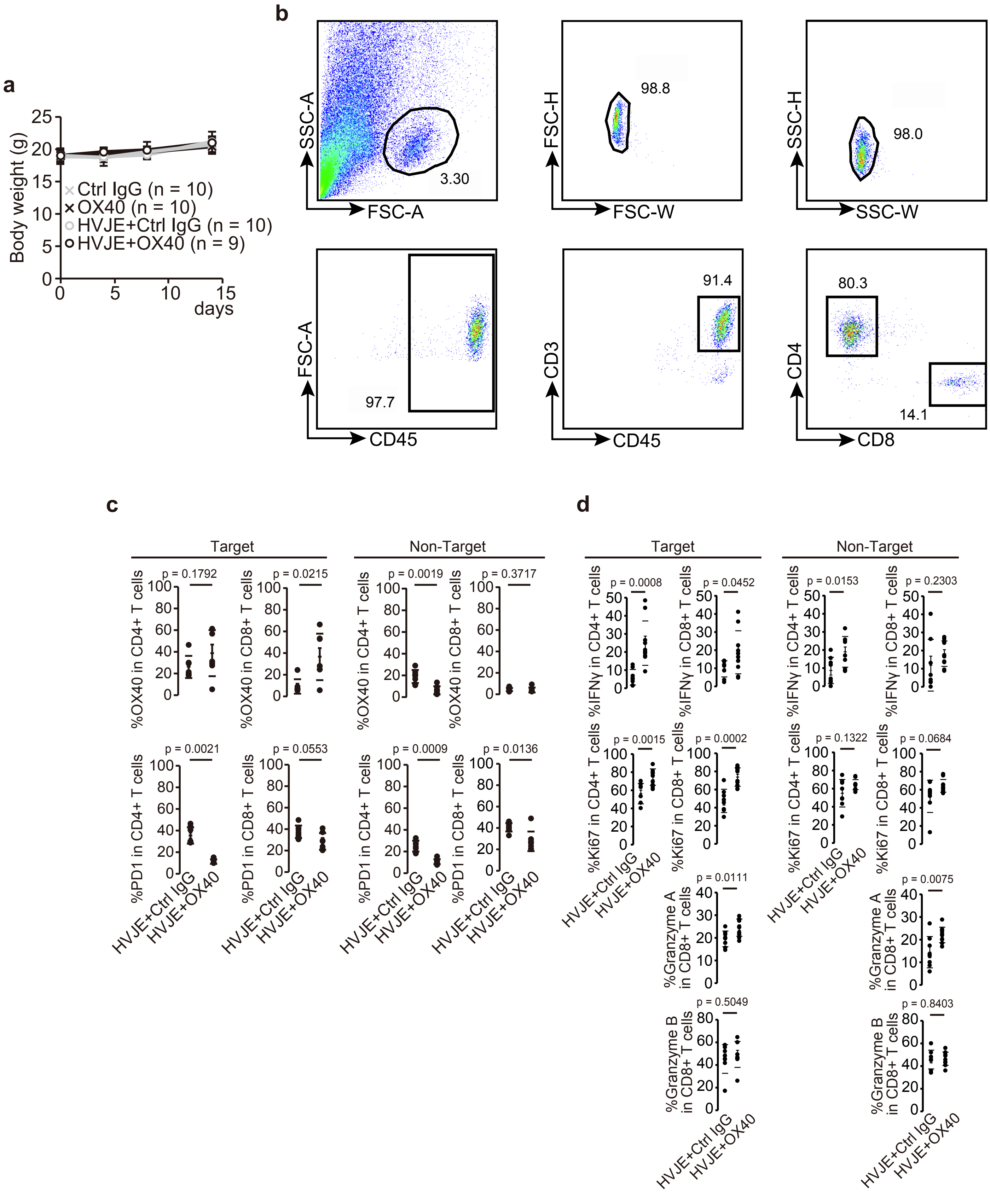
a
Body weight (g)
days
Ctrl IgG (n = 10)
OX40 (n = 10)
HVJE+Ctrl IgG (n = 10)
HVJE+OX40 (n = 9)
b
SSC-A
FSC-A
3.30
FSC-H
FSC-W
98.8
SSC-H
SSC-W
98.0
FSC-A
CD45
97.7
CD3
CD45
91.4
CD4
CD8
80.3
14.1
c
Target
Non-Target
%OX40 in CD4+ T cells
%OX40 in CD8+ T cells
%PD1 in CD4+ T cells
%PD1 in CD8+ T cells
p = 0.1792
p = 0.0215
p = 0.0019
p = 0.3717
p = 0.0021
p = 0.0553
p = 0.0009
p = 0.0136
HVJE+Ctrl IgG
HVJE+OX40
d
Target
Non-Target
%IFNγ in CD4+ T cells
%IFNγ in CD8+ T cells
p = 0.0008
p = 0.0452
p = 0.0153
p = 0.2303
%Ki67 in CD4+ T cells
%Ki67 in CD8+ T cells
p = 0.0015
p = 0.0002
p = 0.1322
p = 0.0684
%Granzyme A in CD8+ T cells
p = 0.0111
p = 0.0075
%Granzyme B in CD8+ T cells
p = 0.5049
p = 0.8403
HVJE+Ctrl IgG
HVJE+OX40

Figure S1; related to Figure 3. Gating strategy of FACS and FACS analysis of the activation of tumor-infiltrating T cells and the body weight of the HVJ-E and anti-OX40 agonist antibody-treated mice. (**a**) Line plot showing the body weight of mice in the different treatment groups. Ctrl, control. Error bars show the SD. (**b**) FACS gating strategy. (**c**) Dot plot of the percentage of OX40- or PD1-positive cells in CD45/CD3/CD4 or CD45/CD3/CD8 positive T cells. HVJ-E (2,000 HAU) was intratumorally injected with 10 μg anti-OX40 agonist or Ctrl antibody on days 0, 2, and 4. Tumors were analyzed 14 days after the initiation of treatment. HVJ-E + Ctrl antibody, n = 7; HVJ-E + anti-OX40 agonist antibody, n = 7 for the target lesion. HVJ-E + Ctrl antibody, n = 8; HVJ-E + anti-OX40 agonist antibody, n = 8 for the non-target lesion. (**d**) Dot plot of the percentage of IFNγ-, Granzyme A-, Granzyme B-, and Ki67-positive cells in CD45+/CD3+/CD4+/Foxp3- and CD45/CD3/CD8 T cells. Tumor-infiltrating lymphocytes were stimulated with PMA and ionomycin in the presence of brefeldin A *ex vivo*. P values were calculated using the Wilcoxon test.

Figure S2

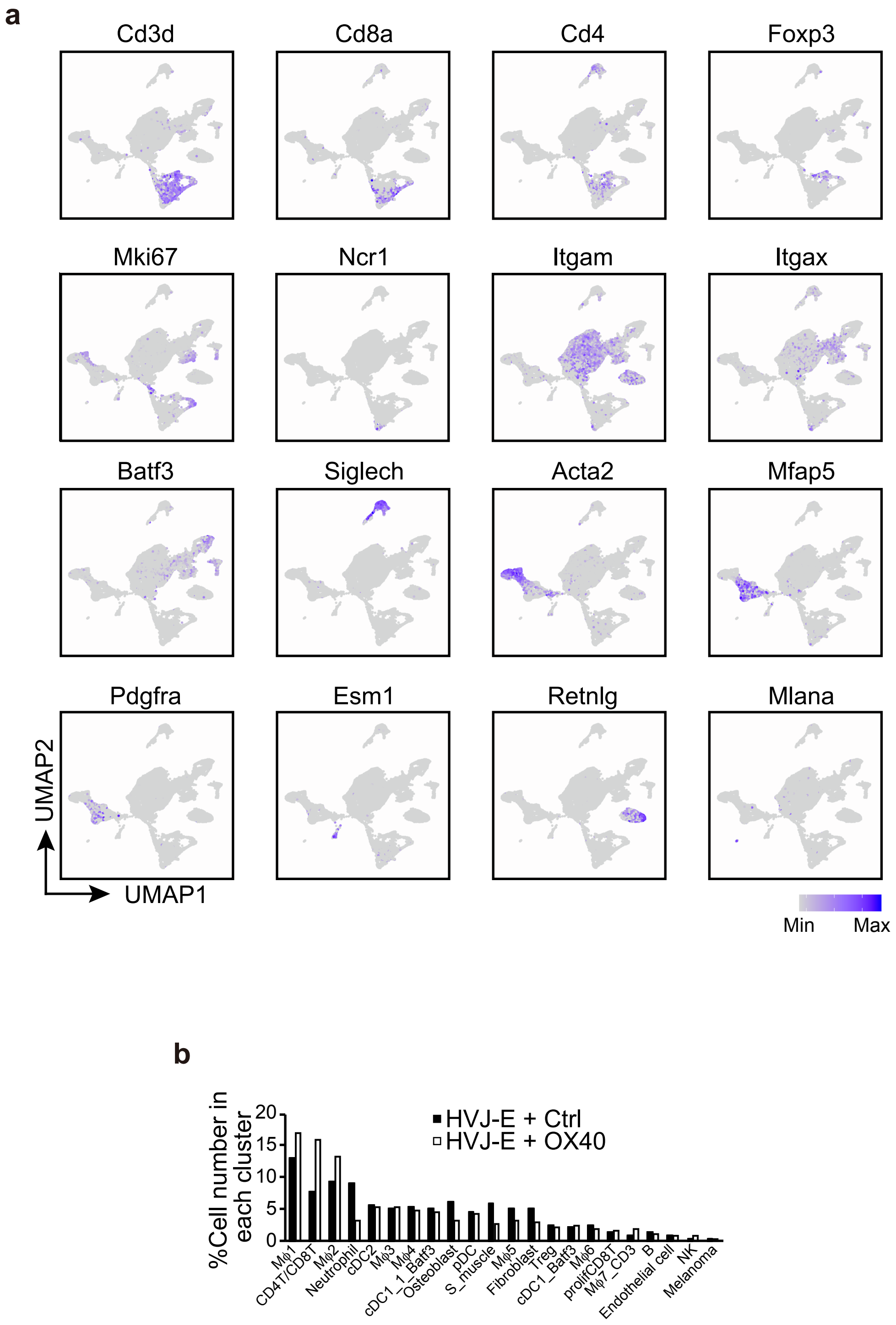
a
Cd3d
Cd8a
Cd4
Foxp3
Mki67
Ncr1
Itgam
Itgax
Batf3
Siglech
Acta2
Mfap5
Pdgfra
Esm1
Retnlg
Mlana
UMAP2
UMAP1
Min
Max
b
%Cell number in each cluster
20
15
10
5
0
HVJ-E + Ctrl
HVJ-E + OX40
Mφ1
CD4T/CD8T
Mφ2
Neutrophil
cDC2
Mφ3
Mφ4
cDC1_1_Batf3
Osteoblast
pDC
S_muscle
Mφ5
Fibroblast
Treg
cDC1_Batf3
Mφ6
prolifCD8T
Mφ7_CD3
B
Endothelial cell
NK
Melanoma

Figure S2; related to Figure 5. Analysis of single-cell RNAseq data of the non-target lesion. (**a**) UMAP of single-cell RNAseq data with the indicated gene expression levels. (**b**) Bar plot of the percentage of cells in each cluster.

Figure S3

a

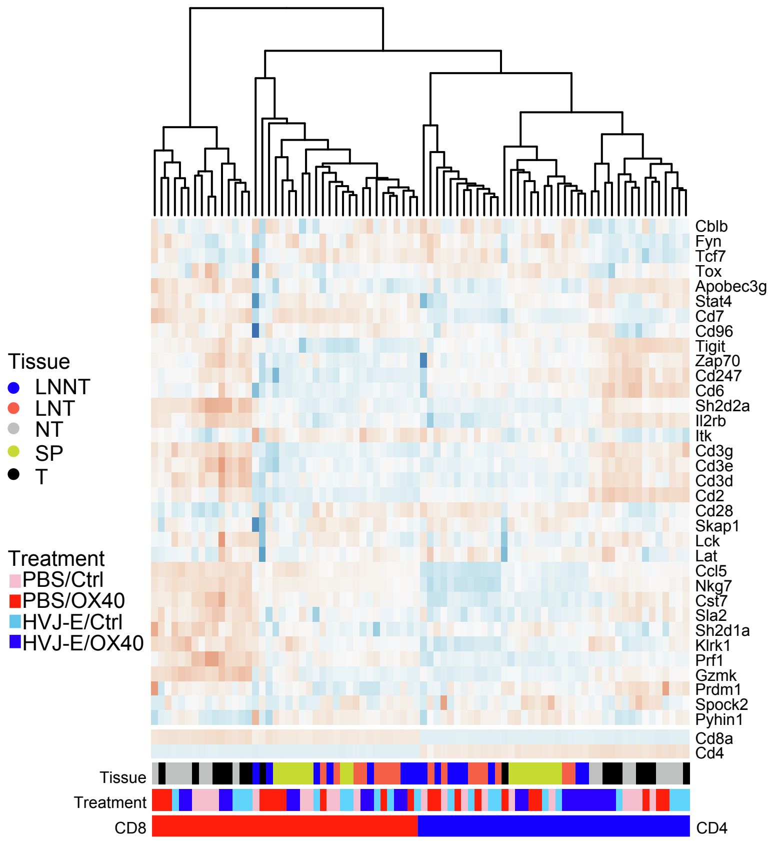

b

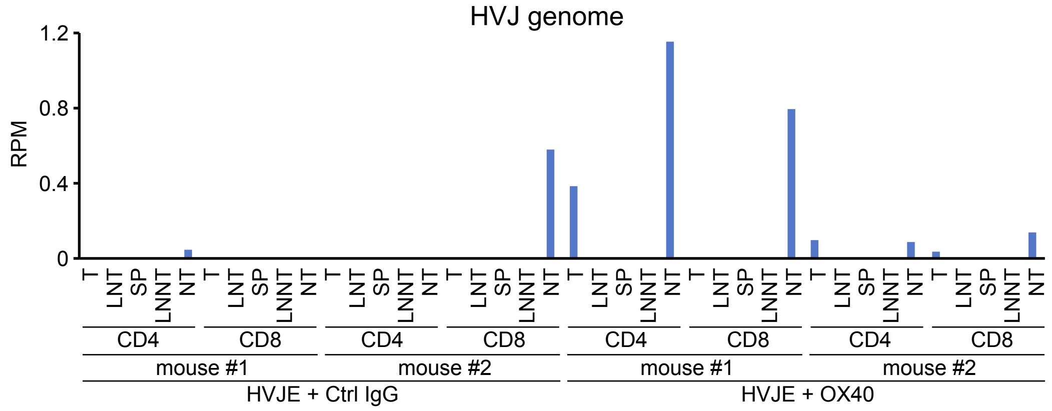

c

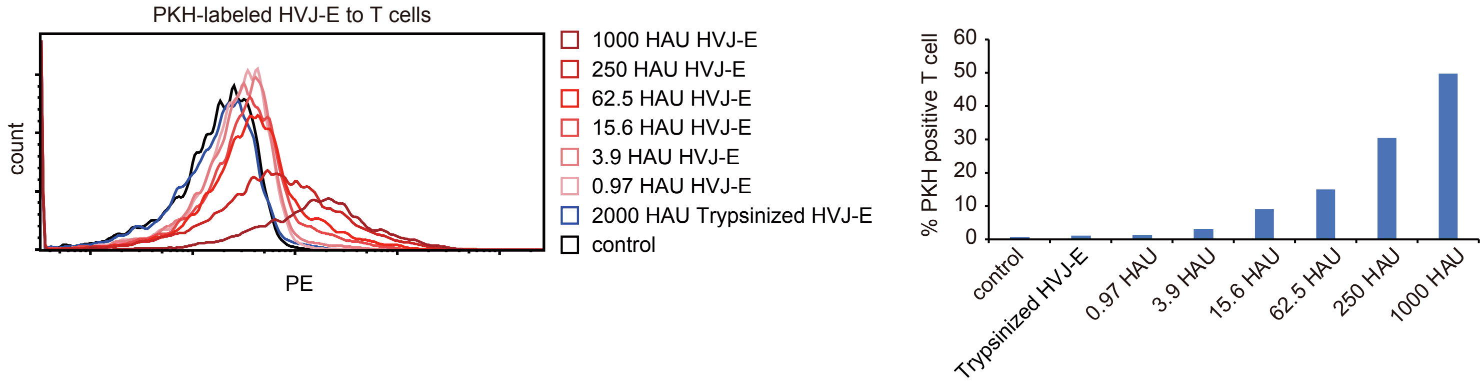

Figure S3; related to Figure 6. Analysis of RNAseq data of CD4 and CD8 T cells at the target and non-target lesions, lymph nodes, and spleen in mice that received HVJ-E + anti-OX40 agonist antibody or control. (**a**) Heat map of T-cell marker genes in CD4 and CD8 T cells. (**b–d**) Dot plot of T cell status scores calculated from RNAseq data of CD4 and CD8 T cells. (**e**) Bar plot of the percentage of HVJ RNA genome in RNAseq data of CD4 and CD8 T cells. (**f**) FACS analysis of infection level of HVJ-E to T cell. PKH-labeled HVJ-E was used to examine the infection level of HVJ-E to T cells. The trypsinized HVJ-E was used as a negative control for staining T cells since the trypsin treatment lost infectivity of HVJ-E to the cell.

Figure S4

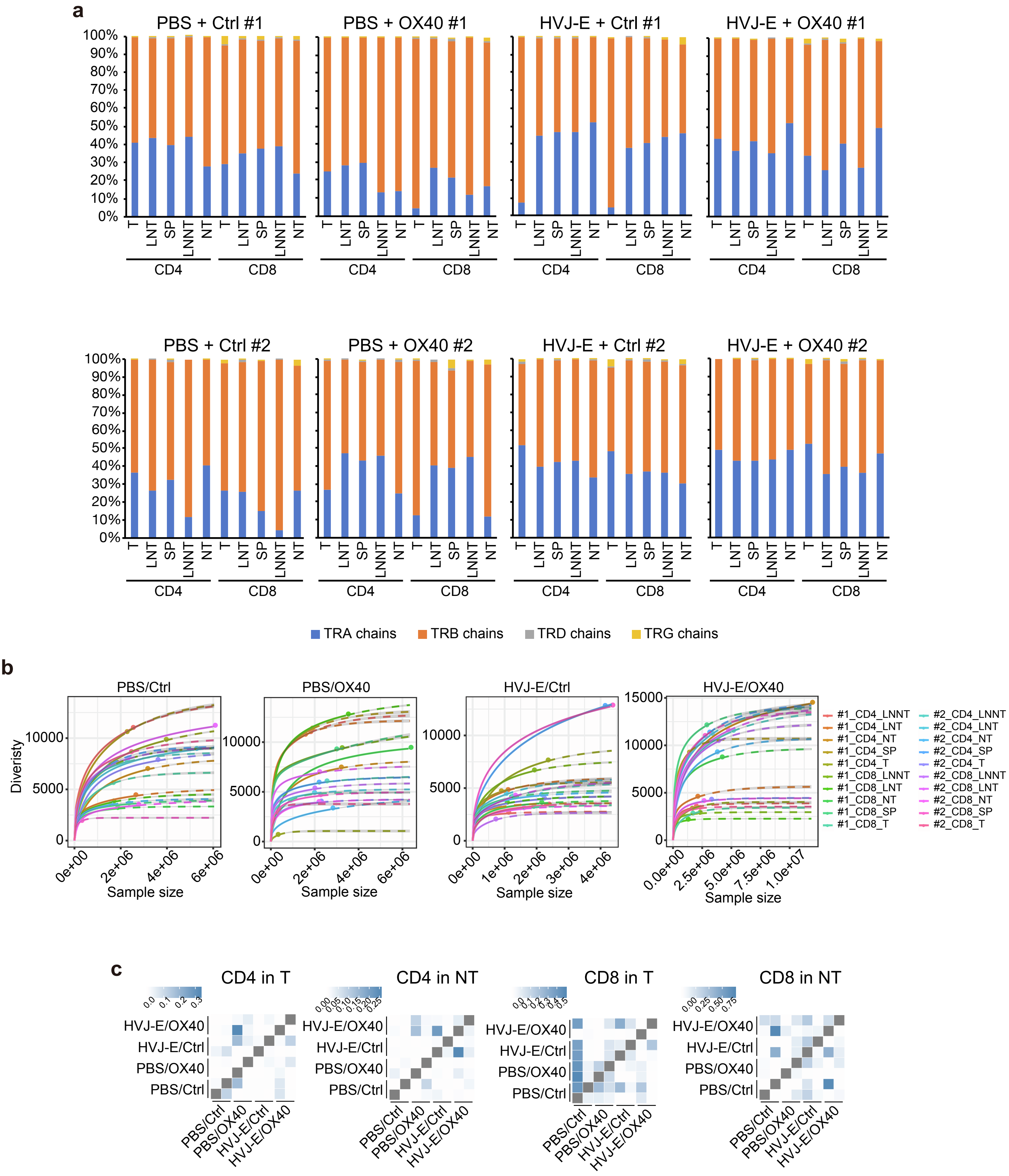
a
PBS + Ctrl #1
PBS + OX40 #1
HVJ-E + Ctrl #1
HVJ-E + OX40 #1
PBS + Ctrl #2
PBS + OX40 #2
HVJ-E + Ctrl #2
HVJ-E + OX40 #2
100%
90%
80%
70%
60%
50%
40%
30%
20%
10%
0%
T
LNT
SP
LNNT
NT
CD4
CD8
TRA chains
TRB chains
TRD chains
TRG chains
b
PBS/Ctrl
PBS/OX40
HVJ-E/Ctrl
HVJ-E/OX40
Diveristy
Sample size
#1_CD4_LNNT
#1_CD4_LNT
#1_CD4_NT
#1_CD4_SP
#1_CD4_T
#1_CD8_LNNT
#1_CD8_LNT
#1_CD8_NT
#1_CD8_SP
#1_CD8_T
#2_CD4_LNNT
#2_CD4_LNT
#2_CD4_NT
#2_CD4_SP
#2_CD4_T
#2_CD8_LNNT
#2_CD8_LNT
#2_CD8_NT
#2_CD8_SP
#2_CD8_T
c
CD4 in T
CD4 in NT
CD8 in T
CD8 in NT
HVJ-E/OX40
HVJ-E/Ctrl
PBS/OX40
PBS/Ctrl

Figure S4; related to Figure 6. Analysis of TCR repertoire of CD4 and CD8 T cells at the target and non-target lesions, lymph nodes, and spleen in mice treated with HVJ-E + anti-OX40 agonist antibody or control. (**a**) Stacked bar plot of TRA, TRB, TRD, and TRG chains in each RNAseq data. (**b**) Rarefaction curve of TCR beta repertoire data showing the dependency between sample diversity and sample size. Dot, current sample size; solid line, interpolated region; dashed line, extrapolated region; shaded area, 95% confidence interval. (**c**) Heat map of clonal overlap frequency of TCR beta in CD4 T cells at the target and non-target lesions (CD4 in T and CD4 in NT) and in CD8 T cells at the target and non-target lesions (CD8 in T and CD8 in NT) between mice.

Figure S5

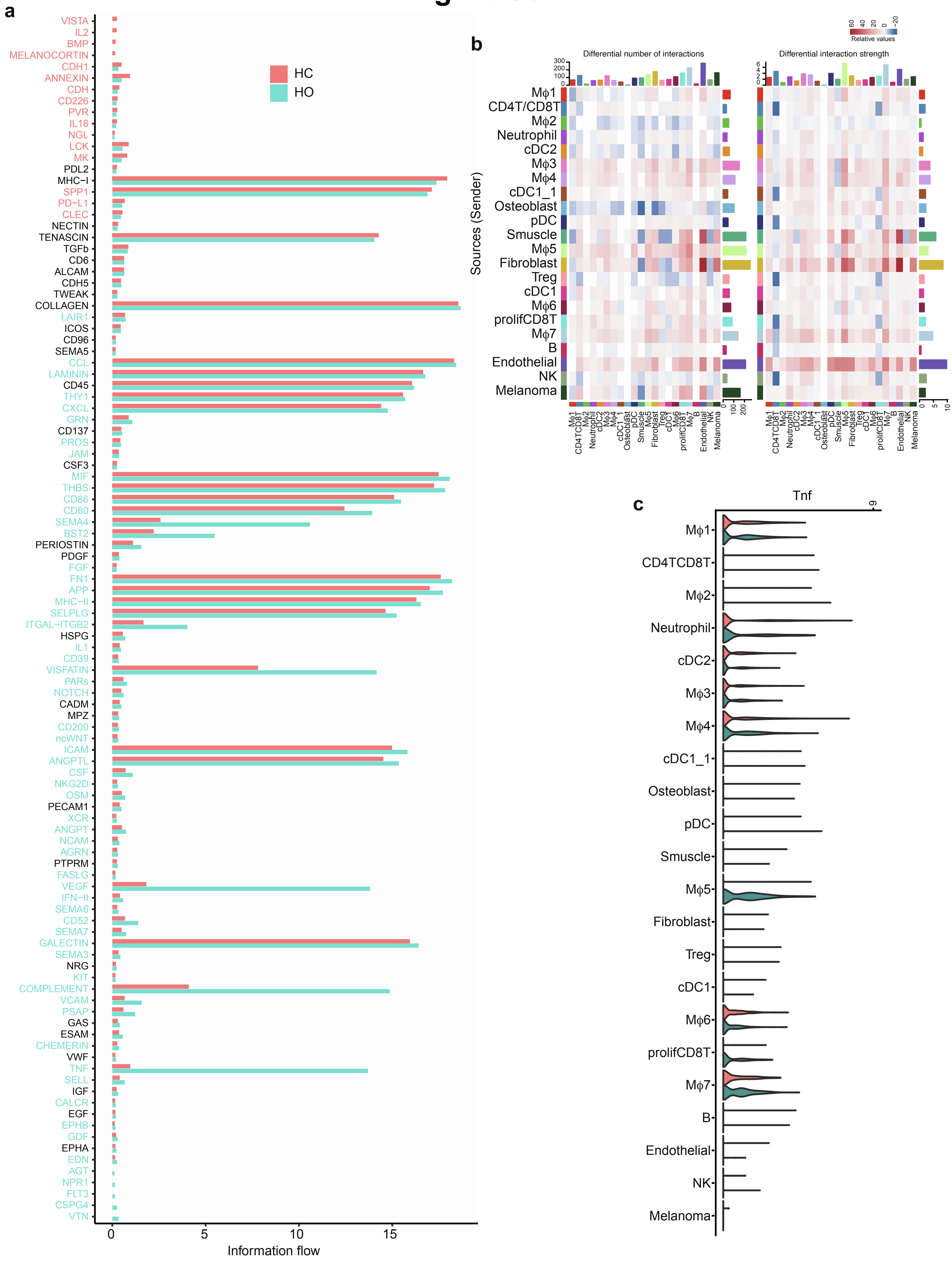
a
HC
HO
VISTA
IL2
BMP
MELANOCORTIN
CDH1
ANNEXIN
CDH
CD226
PVR
IL16
NGL
LCK
MK
PDL2
MHC−I
SPP1
PD−L1
CLEC
NECTIN
TENASCIN
TGFb
CD6
ALCAM
CDH5
TWEAK
COLLAGEN
LAIR1
ICOS
CD96
SEMA5
CCL
LAMININ
CD45
THY1
CXCL
GRN
CD137
PROS
JAM
CSF3
MIF
THBS
CD86
CD80
SEMA4
BST2
PERIOSTIN
PDGF
FGF
FN1
APP
MHC−II
SELPLG
ITGAL−ITGB2
HSPG
IL1
CD39
VISFATIN
PARs
NOTCH
CADM
MPZ
CD200
ncWNT
ICAM
ANGPTL
CSF
NKG2D
OSM
PECAM1
XCR
ANGPT
NCAM
AGRN
PTPRM
FASLG
VEGF
IFN−II
SEMA6
CD52
SEMA7
GALECTIN
SEMA3
NRG
KIT
COMPLEMENT
VCAM
PSAP
GAS
ESAM
CHEMERIN
VWF
TNF
SELL
IGF
CALCR
EGF
EPHB
GDF
EPHA
EDN
AGT
NPR1
FLT3
CSPG4
VTN
0
5
10
15
Information flow
b
Differential number of interactions
Differential interaction strength
Relative values
Sources (Sender)
Mϕ1
CD4T/CD8T
Mϕ2
Neutrophil
cDC2
Mϕ3
Mϕ4
cDC1_1
Osteoblast
pDC
Smuscle
Mϕ5
Fibroblast
Treg
cDC1
Mϕ6
prolifCD8T
Mϕ7
B
Endothelial
NK
Melanoma
c
Tnf
Mϕ1
CD4TCD8T
Mϕ2
Neutrophil
cDC2
Mϕ3
Mϕ4
cDC1_1
Osteoblast
pDC
Smuscle
Mϕ5
Fibroblast
Treg
cDC1
Mϕ6
prolifCD8T
Mϕ7
B
Endothelial
NK
Melanoma

Figure S5; related to Figure 6. The signal pathway and cell-cell communication analysis of scRNAseq data of the non-target lesion. (**a**) Bar plot of information flow in the indicated signal pathway. HC, HVJ-E/Ctrl; HO, HVJ-E/OX40. (**b**) Heatmap of cell-cell communication. The left panel shows a differential number of interactions. The right panel shows differential interaction strength. (**C**) Violin plot of Tnf expression in each cluster.

Figure S6

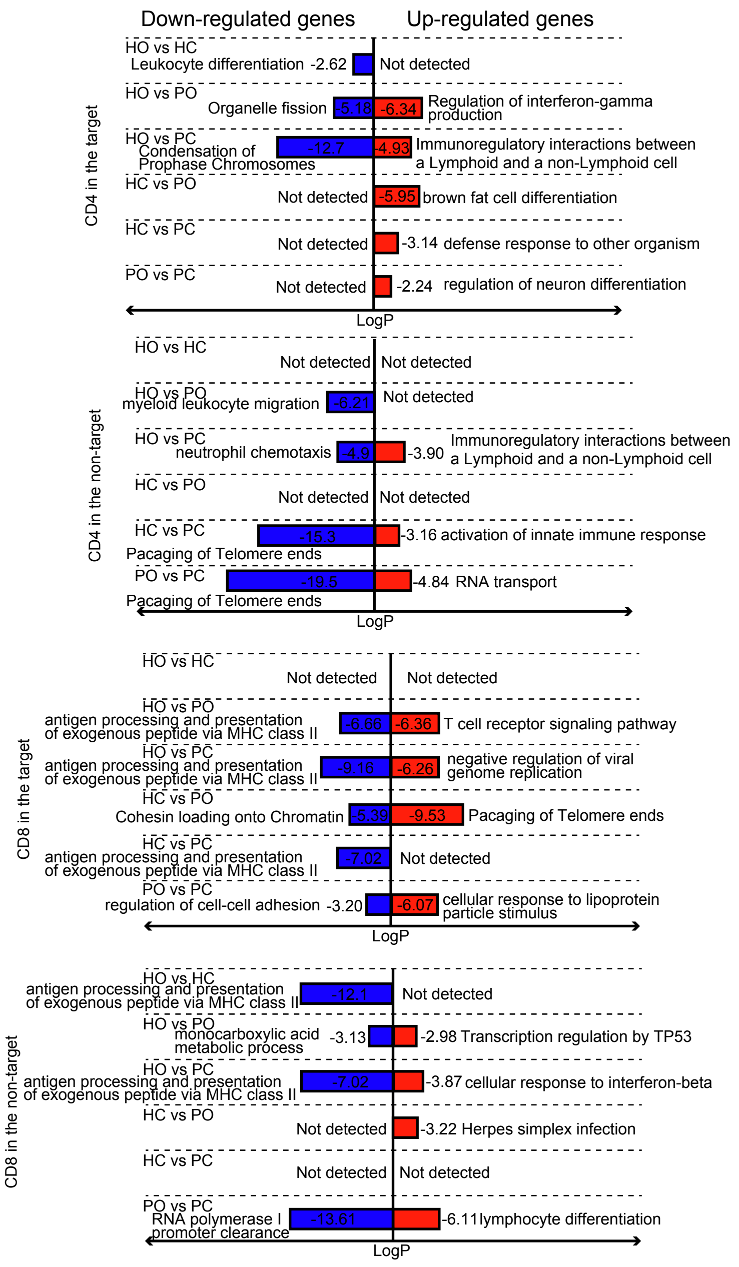

Down-regulated genes
Up-regulated genes
CD4 in the target
HO vs HC
Leukocyte differentiation -2.62
Not detected
HO vs PO
Organelle fission -5.18
-6.34 Regulation of interferon-gamma production
HO vs PC
Condensation of Prophase Chromosomes -12.7
-4.93 Immunoregulatory interactions between a Lymphoid and a non-Lymphoid cell
HC vs PO
Not detected
-5.95 brown fat cell differentiation
HC vs PC
Not detected
-3.14 defense response to other organism
PO vs PC
Not detected
-2.24 regulation of neuron differentiation
LogP
CD4 in the non-target
HO vs HC
Not detected
Not detected
HO vs PO
myeloid leukocyte migration -6.21
Not detected
HO vs PC
neutrophil chemotaxis -4.9
-3.90 Immunoregulatory interactions between a Lymphoid and a non-Lymphoid cell
HC vs PO
Not detected
Not detected
HC vs PC
Pacaging of Telomere ends -15.3
-3.16 activation of innate immune response
PO vs PC
Pacaging of Telomere ends -19.5
-4.84 RNA transport
LogP
CD8 in the target
HO vs HC
Not detected
Not detected
HO vs PO
antigen processing and presentation of exogenous peptide via MHC class II -6.66
-6.36 T cell receptor signaling pathway
HO vs PC
antigen processing and presentation of exogenous peptide via MHC class II -9.16
-6.26 negative regulation of viral genome replication
HC vs PO
Cohesin loading onto Chromatin -5.39
-9.53 Pacaging of Telomere ends
HC vs PC
antigen processing and presentation of exogenous peptide via MHC class II -7.02
Not detected
PO vs PC
regulation of cell-cell adhesion -3.20
-6.07 cellular response to lipoprotein particle stimulus
LogP
CD8 in the non-target
HO vs HC
antigen processing and presentation of exogenous peptide via MHC class II -12.1
Not detected
HO vs PO
monocarboxylic acid metabolic process -3.13
-2.98 Transcription regulation by TP53
HO vs PC
antigen processing and presentation of exogenous peptide via MHC class II -7.02
-3.87 cellular response to interferon-beta
HC vs PO
Not detected
-3.22 Herpes simplex infection
HC vs PC
Not detected
Not detected
PO vs PC
RNA polymerase I promoter clearance -13.61
-6.11 lymphocyte differentiation
LogP

Figure S6; related to Figure 6. Gene ontology (GO) analysis of T cells between treatments: HVJ-E/OX40, HVJ-E/Ctrl, PBS/OX40, and PBS/Ctrl. Representative GO is shown for each comparison. X-axis, log p value. PC, PBS/Ctrl; PO, PBS/OX40; HC, HVJ-E/Ctrl; HO, HVJ-E/OX40.

Figure S7

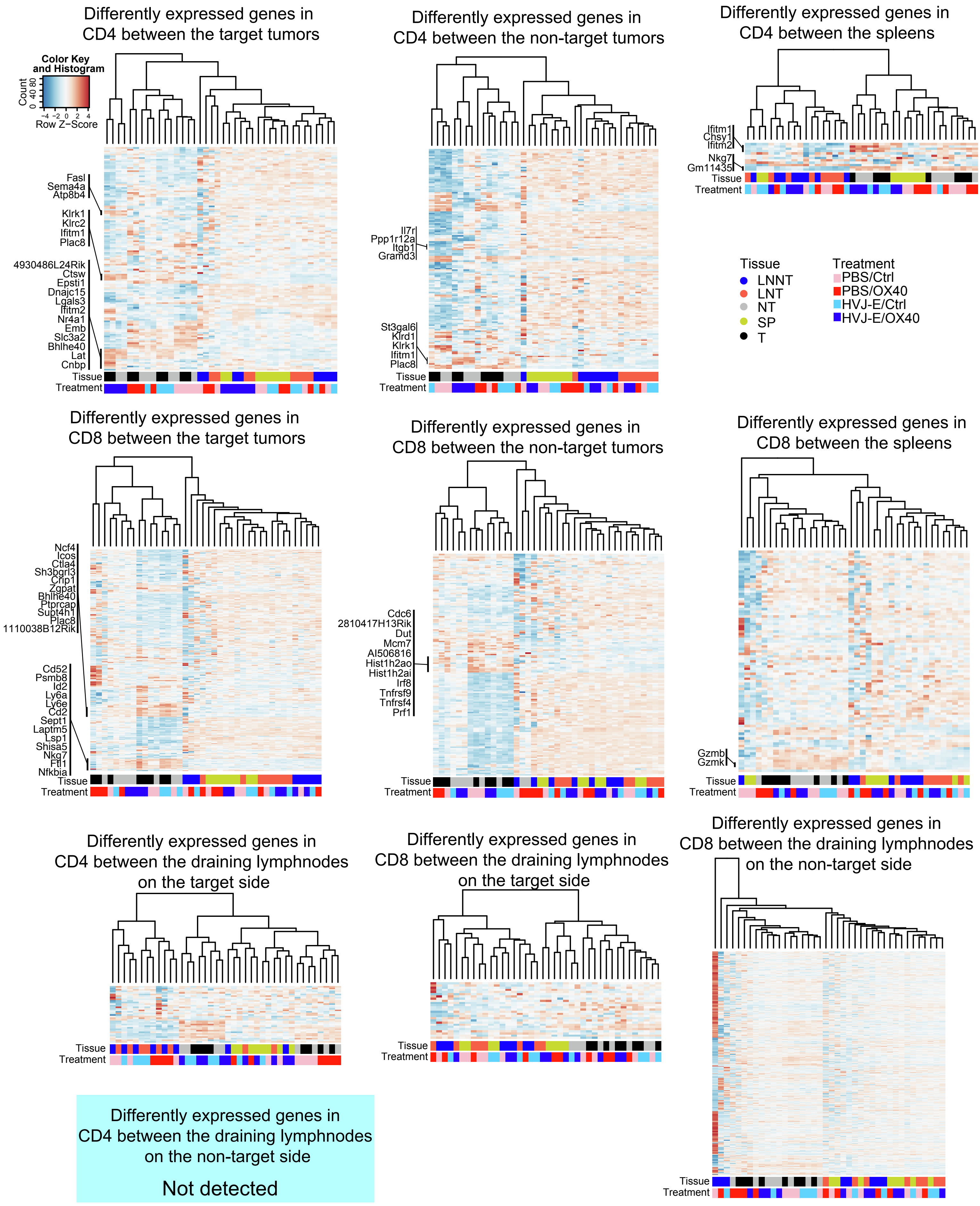
Differently expressed genes in CD4 between the target tumors
Color Key and Histogram
Count
Row Z−Score
Fasl
Sema4a
Atp8b4
Klrk1
Klrc2
Ifitm1
Plac8
4930486L24Rik
Ctsw
Epsti1
Dnajc15
Lgals3
Ifitm2
Nr4a1
Emb
Slc3a2
Bhlhe40
Lat
Cnbp
Tissue
Treatment
Differently expressed genes in CD4 between the non-target tumors
Il7r
Ppp1r12a
Itgb1
Gramd3
St3gal6
Klrd1
Klrk1
Ifitm1
Plac8
Tissue
Treatment
Differently expressed genes in CD4 between the spleens
Ifitm1
Chsy1
Ifitm2
Nkg7
Gm11435
Tissue
Treatment
Tissue
LNNT
LNT
NT
SP
T
Treatment
PBS/Ctrl
PBS/OX40
HVJ-E/Ctrl
HVJ-E/OX40
Differently expressed genes in CD8 between the target tumors
Ncf4
Icos
Ctla4
Sh3bgrl3
Crip1
Zgpat
Bhlhe40
Ptprcap
Supt4h1
Plac8
1110038B12Rik
Cd52
Psmb8
Id2
Ly6a
Ly6e
Cd2
Sept1
Laptm5
Lsp1
Shisa5
Nkg7
Ftl1
Nfkbia
Tissue
Treatment
Differently expressed genes in CD8 between the non-target tumors
Cdc6
2810417H13Rik
Dut
Mcm7
AI506816
Hist1h2ao
Hist1h2ai
Irf8
Tnfrsf9
Tnfrsf4
Prf1
Tissue
Treatment
Differently expressed genes in CD8 between the spleens
Gzmb
Gzmk
Tissue
Treatment
Differently expressed genes in CD4 between the draining lymphnodes on the target side
Tissue
Treatment
Differently expressed genes in CD8 between the draining lymphnodes on the target side
Tissue
Treatment
Differently expressed genes in CD8 between the draining lymphnodes on the non-target side
Tissue
Treatment
Differently expressed genes in CD4 between the draining lymphnodes on the non-target side
Not detected

Figure S7; related to Figure 6. Heat map of T cell gene expression in different treatments: HVJ-E/OX40, HVJ-E/Ctrl, PBS/OX40, and PBS/Ctrl. Genes differentially expressed in T cells from the indicated tissues were selected.

Figure S8

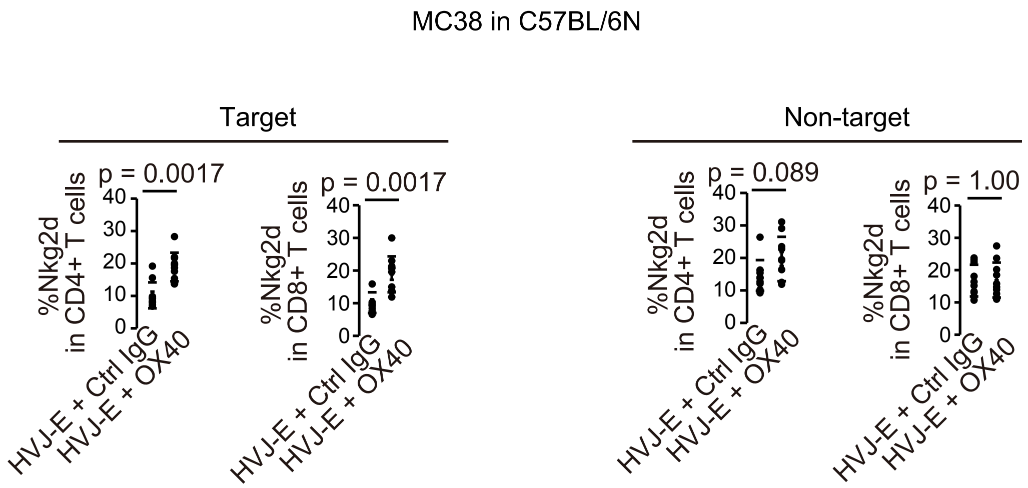
MC38 in C57BL/6N
Target
Non-target
p = 0.0017
p = 0.0017
p = 0.089
p = 1.00
%Nkg2d in CD4+ T cells
%Nkg2d in CD8+ T cells
HVJ-E + Ctrl IgG
HVJ-E + OX40

Figure S8; related to Figure 7. Nkg2d and Cd94 expression in T cells of MC38 bilaterally inoculated mice. Dot plot showing the percentage of Nkg2d and Cd94 expression in CD45/CD3/CD4 and CD45/CD3/CD8 T cells at the target and non-target lesions. MC38 cells (0.5 × 10^6) were bilaterally inoculated into C57BL/6N mice. HVJ-E (2,000 HAU) was intratumorally injected with 10 µg anti-OX40 agonist antibody on days 0, 2, and 4. Tumors were analyzed 14 days after the initiation of treatment. HVJ-E/Ctrl antibody-administrated target tumor, n = 10; HVJ-E/OX40 antibody-administrated target tumor, n = 10; HVJ-E/Ctrl antibody-administrated non-target tumor, n = 10; HVJ-E/OX40 antibody-administrated non-tumor, n = 10. P values were calculated using the Wilcoxon test.

Table S1. Antibody list

For Flow cytometry Analysis

Name(Antigen, Recombinant)	Dye	Reactivity	Catalog no.	Company
CD45	Brilliant Violet 421	Mouse	103134	Biolegend
	APC/Cy7	Mouse	103116	Biolegend
H-2Kd/H-2Dd	Alexa Fluor 647	Mouse	114712	Biolegend
H-2Kd/H-2Dd	PE	Mouse	114708	Biolegend
CD49b	FITC	Mouse	108906	Biolegend
NK1.1	Alexa Fluor 647	Mouse	108720	Biolegend
CD3	FITC	Mouse	100204	Biolegend
	PerCP/Cy5.5	Mouse	100218	Biolegend
	Brilliant Violet 421	Mouse	100228	Biolegend
CD4	APC/Cy7	Mouse	100414	Biolegend
	PerCP/Cy5.5	Mouse	100434	Biolegend
	Alexa Fluor 647	Mouse	100424	Biolegend
CD8a	PE/Cy7	Mouse	100722	Biolegend
	FITC	Mouse	100706	Biolegend
	Brilliant Violet 510	Mouse	100752	Biolegend
	PE	Mouse	100708	Biolegend
CD69	PE	Mouse	104508	Biolegend
OX40	APC	Mouse	119414	Biolegend
PD-1	APC/Cy7	Mouse	135224	Biolegend
CD94	PE/Cy7	Mouse	105510	Biolegend
NKG2D	APC	Mouse	130212	Biolegend
Ki67	PE	Mouse	652404	Biolegend
Ifnγ	PE	Mouse	505808	Biolegend
GranzymeA	PE	Mouse	149704	Biolegend
GranzymeB	PE	Mouse	372208	Biolegend
IA/IE	PE	Mouse	107607	Biolegend
H-2Kb	APC	Mouse	116518	Biolegend
NKG2D-Fc chimera protein			139-NK-050	R&Dsystems
Streptavidin	Brilliant Violet 421		405226	Biolegend

For Animal Experiment

Antibody	Use	Reactivity	Immunogen	Clone	Catalog No.	Company
OX40	Agonist	Mouse	Rat	OX86	119431	Biolegend
PD-1	Blockade	Mouse	Rat	RMP1-14	114108	Biolegend
4-1BB	Agonist	Mouse	Rat	LOB12.3	BE0169	BioXCell
CD8	Depletion	Mouse	Rat	2.43	BE0061	BioXCell
Nkg2d	Blockade	Mouse	Armenian Hamster	HMG2D	BE0111	BioXCell
IgG from Rat Serum	Control				14131	Sigma-Aldrich
Armenian Hamster IgG	Control			HTK888	400902	Biolegend

For Immunohistochemistry

Antibody	Catalog No.	Company
CD4 monoclonal antibody (4SM95)	14-9766-82	eBioscience
CD8a monoclonal antibody (4SM15)	14-0808-82	eBioscience
F4/80 (D2S9R) XP Rabbit mAb	#70076	Cell Signaling Technology
Ki67 Rabbit mAb (SP6)	#418071	Nichirei corporation
Histofine simple stain mouse MAX-PO	#41311	Nichirei corporation
Histofine simple stain MAX-PO	#42141	Nichirei corporation
Anti-Rabbit Immunoglobulins/HRP	P0448	Dako

Table S2. Correlation between replicates of sequencing libraries

Figure	Sample 1	Sample 2	Peason correlation	p value
Figure 6	HVJ-E/Ctrl CD4 LNNT rep1	HVJ-E/Ctrl CD4 LNNT rep2	0.9277318	< 2.2e-16
	HVJ-E/Ctrl CD4 LNT rep1	HVJ-E/Ctrl CD4 LNT rep2	0.9629368	< 2.2e-16
	HVJ-E/Ctrl CD4 NT rep1	HVJ-E/Ctrl CD4 NT rep2	0.9533823	< 2.2e-16
	HVJ-E/Ctrl CD4 SP rep1	HVJ-E/Ctrl CD4 SP rep2	0.9599697	< 2.2e-16
	HVJ-E/Ctrl CD4 T rep1	HVJ-E/Ctrl CD4 T rep2	0.925574	< 2.2e-16
	HVJ-E/Ctrl CD8 LNNT rep1	HVJ-E/Ctrl CD8 LNNT rep2	0.827326	< 2.2e-16
	HVJ-E/Ctrl CD8 LNT rep1	HVJ-E/Ctrl CD8 LNT rep2	0.9107265	< 2.2e-16
	HVJ-E/Ctrl CD8 NT rep1	HVJ-E/Ctrl CD8 NT rep2	0.9282933	< 2.2e-16
	HVJ-E/Ctrl CD8 SP rep1	HVJ-E/Ctrl CD8 SP rep2	0.9633328	< 2.2e-16
	HVJ-E/Ctrl CD8 T rep1	HVJ-E/Ctrl CD8 T rep2	0.9572798	< 2.2e-16
	HVJ-E/OX40 CD4 LNNT rep1	HVJ-E/OX40 CD4 LNNT rep2	0.9565594	< 2.2e-16
	HVJ-E/OX40 CD4 LNT rep1	HVJ-E/OX40 CD4 LNT rep2	0.9695247	< 2.2e-16
	HVJ-E/OX40 CD4 NT rep1	HVJ-E/OX40 CD4 NT rep2	0.9685495	< 2.2e-16
	HVJ-E/OX40 CD4 SP rep1	HVJ-E/OX40 CD4 SP rep2	0.9580791	< 2.2e-16
	HVJ-E/OX40 CD4 T rep1	HVJ-E/OX40 CD4 T rep2	0.9381782	< 2.2e-16
	HVJ-E/OX40 CD8 LNNT rep1	HVJ-E/OX40 CD8 LNNT rep2	0.8790323	< 2.2e-16
	HVJ-E/OX40 CD8 LNT rep1	HVJ-E/OX40 CD8 LNT rep2	0.9418284	< 2.2e-16
	HVJ-E/OX40 CD8 NT rep1	HVJ-E/OX40 CD8 NT rep2	0.9554944	< 2.2e-16
	HVJ-E/OX40 CD8 SP rep1	HVJ-E/OX40 CD8 SP rep2	0.9528603	< 2.2e-16
	HVJ-E/OX40 CD8 T rep1	HVJ-E/OX40 CD8 T rep2	0.9262621	< 2.2e-16
	PBS/Ctrl CD4 LNNT rep1	PBS/Ctrl CD4 LNNT rep2	0.7376137	< 2.2e-16
	PBS/Ctrl CD4 LNT rep1	PBS/Ctrl CD4 LNT rep2	0.8904814	< 2.2e-16
	PBS/Ctrl CD4 NT rep1	PBS/Ctrl CD4 NT rep2	0.9480826	< 2.2e-16
	PBS/Ctrl CD4 SP rep1	PBS/Ctrl CD4 SP rep2	0.9219823	< 2.2e-16
	PBS/Ctrl CD4 T rep1	PBS/Ctrl CD4 T rep2	0.9205372	< 2.2e-16
	PBS/Ctrl CD8 LNNT rep1	PBS/Ctrl CD8 LNNT rep2	0.6342419	< 2.2e-16
	PBS/Ctrl CD8 LNT rep1	PBS/Ctrl CD8 LNT rep2	0.9335547	< 2.2e-16
	PBS/Ctrl CD8 NT rep1	PBS/Ctrl CD8 NT rep2	0.9471465	< 2.2e-16
	PBS/Ctrl CD8 SP rep1	PBS/Ctrl CD8 SP rep2	0.8229628	< 2.2e-16
	PBS/Ctrl CD8 T rep1	PBS/Ctrl CD8 T rep2	0.9265479	< 2.2e-16
	PBS/OX40 CD4 LNNT rep1	PBS/OX40 CD4 LNNT rep2	0.8307282	< 2.2e-16
	PBS/OX40 CD4 LNT rep1	PBS/OX40 CD4 LNT rep2	0.8507964	< 2.2e-16
	PBS/OX40 CD4 NT rep1	PBS/OX40 CD4 NT rep2	0.8818254	< 2.2e-16
	PBS/OX40 CD4 SP rep1	PBS/OX40 CD4 SP rep2	0.9357758	< 2.2e-16
	PBS/OX40 CD4 T rep1	PBS/OX40 CD4 T rep2	0.8937389	< 2.2e-16
	PBS/OX40 CD8 LNNT rep1	PBS/OX40 CD8 LNNT rep2	0.7960244	< 2.2e-16
	PBS/OX40 CD8 LNT rep1	PBS/OX40 CD8 LNT rep2	0.8493413	< 2.2e-16
	PBS/OX40 CD8 NT rep1	PBS/OX40 CD8 NT rep2	0.8189762	< 2.2e-16
	PBS/OX40 CD8 SP rep1	PBS/OX40 CD8 SP rep2	0.8551301	< 2.2e-16
	PBS/OX40 CD8 T rep1	PBS/OX40 CD8 T rep2	0.8213812	< 2.2e-16

Table S3. Supplemental table for bioinformatic tools

tool	aim
bedtools	Data formating
bowtie2	Mapping reads to genome
Cell Ranger	Single cell analysis
CellChat	Single cell analysis
CIBERSORTx	Deconvolution of RNAseq data
clusterProfiler	Analysis of gene ontology
DESeq2	RNAseq analysis
DOSE	Analysis of gene ontology
enrichplot	Analysis of gene ontology
FastQC	Check data quality
ggplot2	Draw figures
ggrepel	Draw figures
gplots	Draw heatmap
IGV	Draw sequencing tracks
IGVtools	Data formating
MIXCR	Analysis of TCR reperitore
org.Mm.eg.db	Analysis of gene ontology
R 3.6.3	R programing
Rstudio	R programing
Rstudio Server	R programing
Samtools	Data formating
Seurat	Single cell analysis
STAR	Mapping reads to genome
Stringtie	RNAseq analysis
VDJtools	Analysis of TCR reperitore
